# A specific molecular signature in SARS-CoV-2–infected kidney biopsies

**DOI:** 10.1172/jci.insight.165192

**Published:** 2023-03-08

**Authors:** Pierre Isnard, Paul Vergnaud, Serge Garbay, Matthieu Jamme, Maeva Eloudzeri, Alexandre Karras, Dany Anglicheau, Valérie Galantine, Arwa Jalal Eddine, Clément Gosset, Franck Pourcine, Mohammed Zarhrate, Jean-Baptiste Gibier, Elena Rensen, Stefano Pietropaoli, Giovanna Barba-Spaeth, Jean-Paul Duong-Van-Huyen, Thierry J. Molina, Florian Mueller, Christophe Zimmer, Marco Pontoglio, Fabiola Terzi, Marion Rabant

**Affiliations:** 1 University of Paris Cité, INSERM U1151, CNRS UMR 8253, Institut Necker-Enfants Malades, Département Croissance et Signalisation, Paris, France.; 2 Department of Pathology, Centre Hospitalier Universitaire Necker-Enfants Malades, Assistance Publique – Hopitaux de Paris (AP-HP), Paris, France.; 3 Department of Intensive Care Medicine, Centre Hospitalier Intercommunal de Poissy, Poissy, France.; 4 Department of Nephrology, Centre Hospitalier Universitaire Européen Georges Pompidou, Paris, France.; 5 Department of Transplantation, Centre Hospitalier Universitaire Necker-Enfants Malades, Paris, France.; 6 Department of Nephrology, Centre Hospitalier Universitaire de la Guadeloupe, Pointe-à-Pitre, France.; 7 Department of Nephrology, Hôpital Foch, Paris, France.; 8 Department of Nephrology, Centre Hospitalier Universitaire de La Réunion, Saint Denis de La Réunion, France.; 9 Department of Nephrology, Centre Hospitalier de Melun, Melun, France.; 10 Genomics Core Facility, Structure Fédérative de Recherche Necker, University of Paris, Paris, France.; 11 Department of Pathology, Centre Hospitalier Universitaire (CHU) Lille, Lille, France.; 12 Imaging and Modeling Unit and; 13 Structural Virology Unit, Institut Pasteur, Paris, France.

**Keywords:** COVID-19, Nephrology, Apoptosis, Molecular pathology

## Abstract

Acute kidney injury is one of the most important complications in patients with COVID-19 and is considered a negative prognostic factor with respect to patient survival. The occurrence of direct infection of the kidney by SARS-CoV-2, and its contribution to the renal deterioration process, remain controversial issues. By studying 32 renal biopsies from patients with COVID-19, we verified that the major pathological feature of COVID-19 is acute tubular injury (ATI). Using single-molecule fluorescence in situ hybridization, we showed that SARS-CoV-2 infected living renal cells and that infection, which paralleled renal angiotensin-converting enzyme 2 expression levels, was associated with increased death. Mechanistically, a transcriptomic analysis uncovered specific molecular signatures in SARS-CoV-2–infected kidneys as compared with healthy kidneys and non–COVID-19 ATI kidneys. On the other hand, we demonstrated that SARS-CoV-2 and hantavirus, 2 RNA viruses, activated different genetic networks despite triggering the same pathological lesions. Finally, we identified X-linked inhibitor of apoptosis-associated factor 1 as a critical target of SARS-CoV-2 infection. In conclusion, this study demonstrated that SARS-CoV-2 can directly infect living renal cells and identified specific druggable molecular targets that can potentially aid in the design of novel therapeutic strategies to preserve renal function in patients with COVID-19.

## Introduction

Coronavirus disease 2019 (COVID-19) is a recently discovered β-subtype coronavirus infection due to SARS coronavirus 2 (SARS-CoV-2) (1). COVID-19 has enormous health, economic, and social impacts, resulting in a major global public issue. In the majority of cases, patients exhibit mild symptoms. However, in more severe cases, patients present an acute respiratory disease with interstitial and alveolar pneumonia, which can lead to respiratory failure requiring mechanical ventilation (2). Acute kidney injury (AKI) is one of the most important complications in patients with COVID-19, occurring in almost 10% of all cases and around 50% among hospitalized patients (3–6). Despite improved knowledge and patient management, which led to a significant reduction in mortality after the first pandemic wave, AKI remains a major complication in patients with COVID-19 (7). More importantly, AKI is considered a negative prognostic factor with respect to disease severity and patient survival (6, 8, 9). Patients with COVID-19 also frequently present biological evidence of renal dysfunction, such as proteinuria, hematuria, or manifestations of proximal tubule impairment (3, 10–12).

Anatomo-pathological studies revealed that COVID-19–associated kidney disease results in 2 major morphological findings: acute tubular injury (ATI) and collapsing glomerulopathy (CG) (13, 14). CG has emerged as a distinct pathology and appears to affect mainly patients of African ancestry who have a high-risk apolipoprotein L1 (*APOL1*) genotype (15, 16).

Several potential mechanisms have been proposed to explain COVID-19–associated kidney disease, including direct cytokine or complement-mediated injury, coagulopathy, endothelial cell dysfunction, and ischemic/hypoxic processes (17–19). However, the precise pathophysiology mechanisms leading to kidney lesions are not yet elucidated. Thus, there is an urgent need to uncover the molecular pathways underlying these pathological changes to develop preventive strategies.

Currently, it is still controversial whether SARS-CoV-2 can directly infect the kidney (20). Although a recent publication has shown a direct SARS-CoV-2 infection of kidney epithelial cells (21), several other studies argue against this result (13, 22–26). For example, May et al. have recently failed to detect direct viral infection using immunohistochemistry in a large series of 240 native kidneys and 44 allografts (27). Along these lines, the identification of viral particles by electron microscopy (EM) or immunohistochemistry (IHC) has been challenged (28–30). Indeed, viral particles may be confused with normal cell organelles such as clathrin-coated vesicles or multivesicular bodies in EM. On the other hand, the absence of appropriate negative controls prevents any definitive conclusion in the immunohistochemical studies. The presence of viral particles in kidney and other organs using in situ hybridization has been mainly shown in material from autopsies. However, in this context, we cannot exclude that the multiorgan failure and severe SARS-CoV-2 infection in patients prior to death may favor virus diffusion, raising the question of whether SARS-CoV-2 really infects living renal cells and triggers lesions.

Here, we used cutting-edge technologies to characterize the pathophysiology of kidney injury in a potentially unique, large kidney biopsy series of living SARS-CoV-2–infected patients collected at the time of kidney damage, including patients undergoing mechanical ventilation in an intensive care unit. From March 2020 to March 2021, we prospectively enrolled 32 consecutive patients with COVID-19. Our results validated the 3 main pathological changes most frequently reported in kidneys and identified a likely new morphological entity. In addition, we clearly showed SARS-CoV-2 infected living renal cells and that this infection correlated with the level of angiotensin-converting enzyme 2 (ACE2), the cellular receptor for SARS-CoV-2 entry. More importantly, we identified specific molecular signatures in SARS-CoV-2–infected kidneys that may provide a basis for the development of targeted therapeutic strategies.

## Results

### Clinical characteristics.

The demographic characteristics and clinical and biological data of the 32 patients with COVID-19 and controls are provided in Table 1, Supplemental Tables 1–3, and Supplemental Figure 1A; supplemental material available online with this article; https://doi.org/10.1172/jci.insight.165192DS1 Briefly, patients had a mean age of 56 years (range, 4–83 years), and the majority were males (sex ratio, 2.6). Twenty-eight patients underwent native kidney biopsies, and 4 had allograft specimens. Frequent and notable comorbidities included diabetes (7 patients, 22%), obesity (6 patients, 19%), high blood pressure (19 patients, 59%), and chronic kidney disease (8 patients, 25%). SARS-CoV-2 infection was confirmed by reverse transcription PCR (RT-PCR) for 29 patients, and SARS-CoV-2 antibodies were detected in 3 unvaccinated patients. Twenty-four patients (75%) had COVID-19 pneumonia, including 14 patients (44%) with a severe form requiring mechanical ventilation. Twenty-six patients (81%) displayed AKI, of whom 16 (50%) required dialysis. The other indication for renal biopsy was the appearance of proteinuria in 6 patients (19%). The delay between the onset of COVID-19 and renal biopsy was on average 28 days (range, 3–87 days). Eight patients (25%) eventually died from multiorgan failure.

### Renal morphological findings.

A careful morphological analysis of kidney biopsies (Supplemental Figure 1 and Supplemental Table 4) revealed 3 main patterns of kidney-associated COVID-19 disease: ATI in 14 patients (44%; Supplemental Figure 1B), CG with ATI in 10 patients (31%; Supplemental Figure 1C), and thrombotic microangiopathy with C3 glomerulonephritis (TMA-C3GN) in 4 patients (12.5%; Supplemental Figure 1D). Other pathological features included diabetic nephropathy or focal segmental glomerulosclerosis with severe interstitial fibrosis in 4 patients (12.5%).

Pure ATI lesions were mainly observed in patients with more severe COVID-19 (Table 1 and Supplemental Figure 1A) and were histologically comparable to 6 non–COVID-19 ATI except for more frequent polymorphonuclear cells in peritubular capillaritis in patients with COVID-19 (Supplemental Table 5). Consistent with previous reports (14–16), patients with combined CG and ATI were all of African ancestry, and all tested patients (*n* = 6) had an *APOL1* high-risk gene variant.

Interestingly, 4 patients showed the association of features of endocapillary proliferative glomerulonephritis (GN), enriched in polynuclear cells, resembling postinfectious GN, with glomerular and/or arteriolar TMA (Supplemental Figure 1D and Supplemental Tables 6 and 7). Of note, subepithelial humps were observed in only 1 patient (patient 26). Importantly, complement C3 and/or C4 serum levels were decreased in 3 of the 4 patients, suggesting an activation of the complement pathway.

### SARS-CoV-2 infects kidney cells.

**Figure 1:**
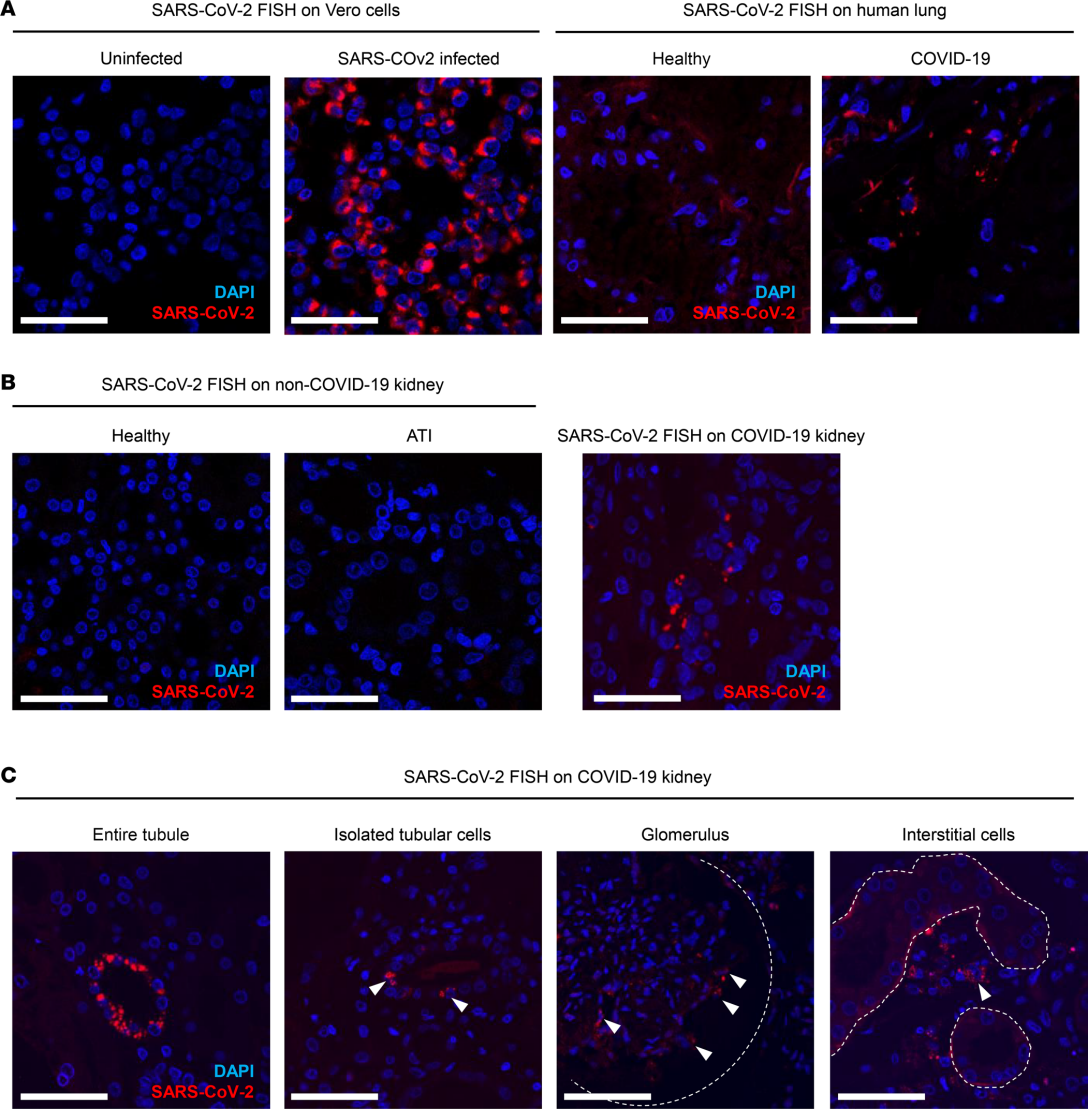
SARS-CoV-2 infects kidney cells. (**A**) Results of SARS-CoV-2 FISH on uninfected and SARS-CoV-2–infected Vero cells (left) and on human lung tissue of healthy and COVID-19 patients (right). (**B**) Results of SARS-CoV-2 FISH on control kidneys (healthy and ATI of non–COVID-19 patients) and on a kidney from a patient with COVID-19. (**C**) Representative images of the different patterns of SARS-CoV-2 FISH detection in kidneys from patients with COVID-19. Of note, the first 2 images on the left show SARS-CoV-2 staining in tubules. The third image from the left shows SARS-CoV-2 staining in a glomerulus. The dotted lines outline the glomerular capsule. The fourth image (right) shows SARS-CoV-2 staining in interstitial cells. The dotted lines outline the tubular basal membrane. White arrows show positive cells. Positive-strand RNA was labeled with Cy5 (red), and nuclei in blue (DAPI). Scale bars in all panels: 100 μm.

To definitively determine if SARS-CoV-2 infects renal cells, we took advantage of a sensitive single-molecule fluorescence in situ hybridization (smFISH) technique designed to specifically detect SARS-CoV-2 RNA (CoronaFISH) and applied it to our samples from 32 patients with COVID-19 and controls. We detected strong signals characterized by intense cytoplasmic perinuclear dots in formalin-fixed, paraffin-embedded (FFPE) SARS-CoV-2–infected Vero cells and in lung tissue from patients with COVID-19. On the other hand, we did not detect any signal in control FFPE uninfected Vero cells, normal lung tissue, normal kidney tissue, and non–COVID-19 ATI kidneys, validating the specificity of the CoronaFISH approach (Figure 1, A and B). CoronaFISH revealed that SARS-CoV-2 RNA was present in kidneys of 16 patients with COVID-19 (50%) (Figure 1, B and C). The viral RNA was always detected in tubular cells; in some tubules, all cells were positive, whereas in others, only a few cells were stained (Figure 1C). Additionally, 1 patient with CG lesions (patient 17) showed positive interstitial macrophage-like cells, and another (patient 22) displayed positive podocyte-like glomerular cells (Figure 1C). Interestingly, SARS-CoV-2 RNA was detected mainly in kidneys from patients with severe COVID-19 disease and pure ATI lesions (11 patients, 78%, Supplemental Table 4). Moreover, we also detected viral RNA in 5 patients with CG-ATI (50%). It is worth noting that we could not detect any viral RNA in patients with TMA/C3GN and in the “other lesions” group (Supplemental Table 4). There was no difference in the delay of renal biopsy between CoronaFISH-positive and CoronaFISH-negative patients (31 days [range: 3–77 days] compared to 24 days [range: 8–59 days], respectively). However, the mortality rate was significantly higher in COVID-19 patients with SARS-CoV-2–infected kidney (50% versus 7%, Supplemental Table 8). Together, these results show that SARS-CoV-2 can infect renal cells in living patients and that the presence of the virus in renal tubular cells may aggravate the evolution of kidney damage. We next verified viral infection of kidney epithelial cells at the protein level, by performing immunohistochemical staining for the SARS-CoV-2 nucleocapsid protein. As expected, we could not detect any signal in control FFPE uninfected Vero cells and normal lung and kidney tissue, whereas a strong signal was present in FFPE SARS-CoV-2–infected Vero cells and in lung tissue from patients with COVID-19 (Supplemental Figure 2A). Similarly, kidney tissue from patients with COVID-19 was positive for capsid staining. However, kidneys from non–COVID-19 patients with ATI also displayed similar tubular patterns (Supplemental Figure 2, B and C). Together, these data suggest that damaged renal tubules may express antigens that mimic SARS-CoV-2 antigens, making the specificity of SARS-CoV-2 antibodies questionable.

### ACE2 expression levels correlate with renal SARS-CoV-2 infection.

It is known that ACE2, the receptor of SARS-CoV-2, is mostly expressed in renal epithelial cells (31–33). Hence, we examined the ACE2 expression pattern in our SARS-CoV-2–positive and –negative kidneys. We first validated that ACE2 was mainly expressed in cortical tubular cells and, to a lesser extent, in glomerular cells (parietal epithelial cells and podocytes) (Supplemental Figure 3A). Remarkably, we observed that ACE2 expression was significantly increased in kidneys from FISH-positive COVID-19 patients (*n* = 9) as compared with FISH-negative COVID-19 patients (*n* = 10) (*P* < 0.05) (Supplemental Figure 3B). These results suggest that differences in ACE2 levels may predict the increased susceptibility of SARS-CoV-2 infection in the kidneys of patients with COVID-19.

### SARS-CoV-2 renal infection elicits a specific molecular signature.

**Figure 2:**
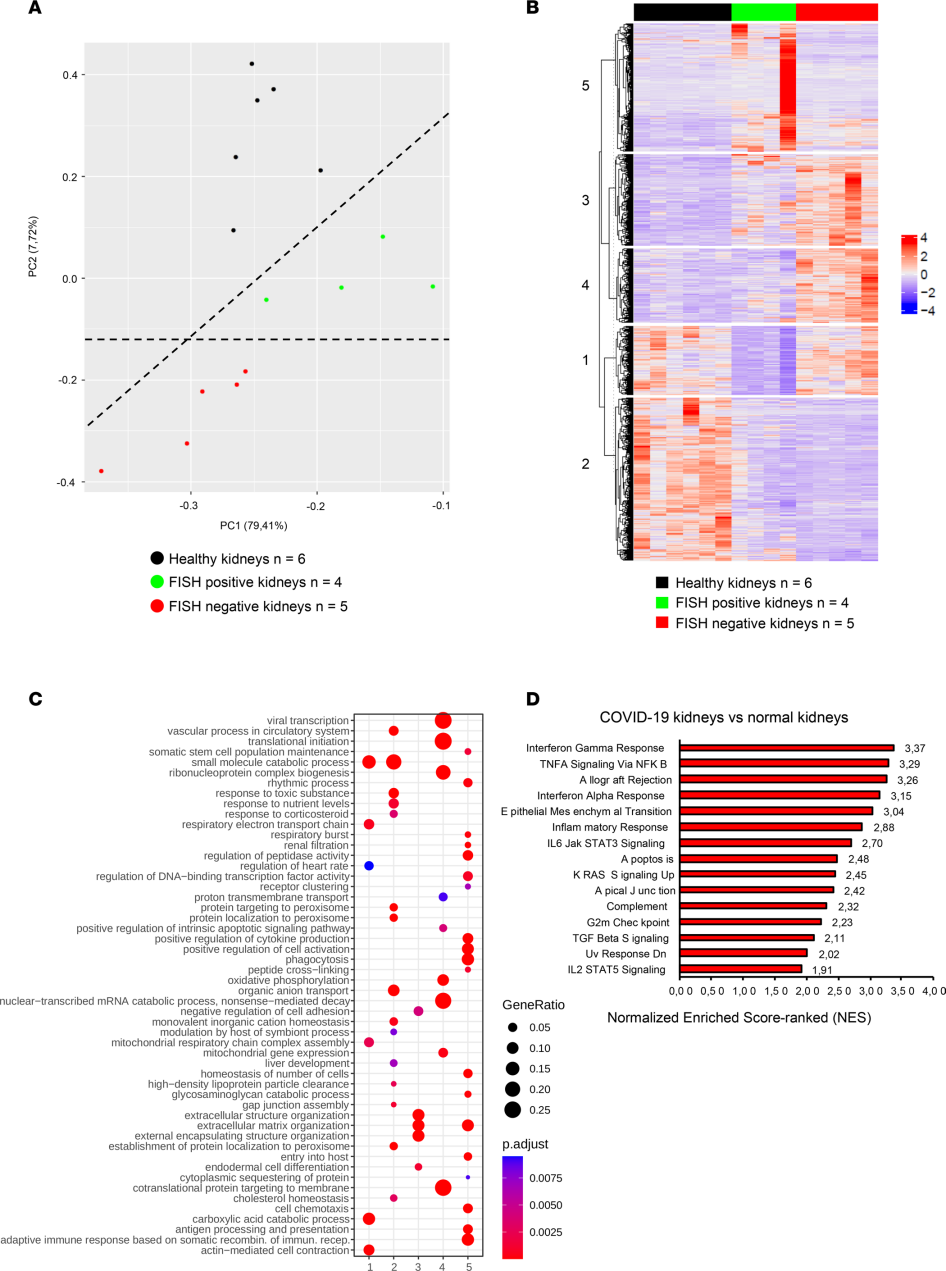
SARS-CoV-2 renal infection elicits a specific molecular signature. (**A**) PCA of the top 500 most variable genes in SARS-CoV-2 FISH-positive and FISH-negative kidneys and healthy kidneys. (**B**) Heatmap representing K-means analysis of DEGs comparing healthy kidneys and SARS-CoV-2 FISH-positive and SARS-CoV-2 FISH-negative COVID-19 kidneys. (**C**) Dot plot of the cluster profiling analysis from K-means comparing healthy kidneys and SARS-CoV-2 FISH-positive and SARS-CoV-2 FISH-negative COVID-19 kidneys. (**D**) Bar plot of the top 15 normalized enriched score–ranked gene sets (hallmark, GSEA) with *q* < 0.001 in COVID-19 kidneys compared with healthy kidneys.

To identify genetic networks that trigger the development of renal lesions during COVID-19, we performed RNA-Seq on damaged kidneys from 9 patients with COVID-19, among whom 4 were positive for SARS-CoV-2 RNA (FISH-positive) and 5 were negative (FISH-negative). Compared with 6 healthy control kidneys, we found 785 differentially expressed genes (DEGs) in COVID-19 samples (Supplemental Figure 4). Principal component analysis (PCA) of the top 500 most variable genes demonstrated that gene expression differences could be driven by 2 main components (Figure 2A). The main predictor of renal gene expression was COVID-19 status, and the second predictor was the presence of renal SARS-CoV-2 RNA, with a sharp separation between FISH-positive and FISH-negative kidneys. Of interest, the analysis of DEGs identified several (K-means) clusters of genes that revealed a peculiar specific pattern underlying the FISH positivity status of kidneys. In particular, the comparison of FISH-positive versus FISH-negative kidneys revealed an increase in expression of specific gene clusters related to immune response pathways and cell–extracellular matrix interactions (Figure 2, B and C).

To determine the most relevant pathways modulated by COVID-19, we performed a gene set enrichment analysis (GSEA) using the “hallmark” gene set (Figure 2D). A significant number of genes related to inflammation, such as IFN-γ and -α responses; TNFA, IL-6, and IL-2 pathways; inflammatory response; and allograft rejection, were highly enriched in COVID-19 kidneys compared with healthy controls. Consistent with the pathogenesis of COVID-19, complement and apoptosis pathways were also specifically enriched in COVID-19 kidneys. A significant number of genes related to the G2/M cell cycle checkpoint were also upregulated in COVID-19 kidneys.

**Figure 3:**
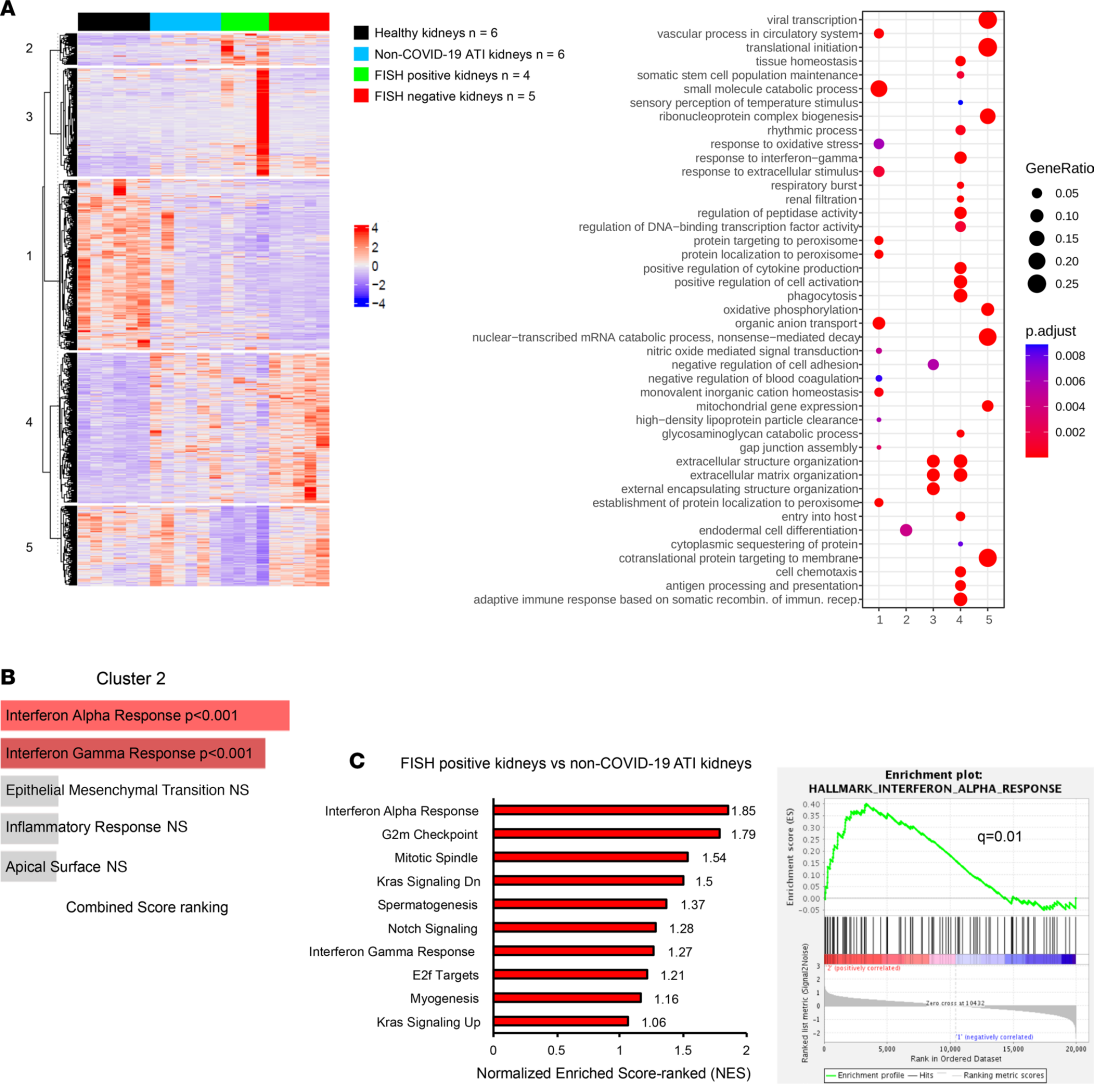
SARS-CoV-2 renal infection elicits a specific molecular signature compared with non–COVID-19 ATI kidneys. (**A**) Heatmap representing K-means analysis with the corresponding dot plot of the cluster profiling of DEGs comparing healthy kidneys, SARS-CoV-2 FISH-positive, SARS-CoV-2 FISH-negative, and non–COVID-19 ATI kidneys. (**B**) Bar plot of the top 5 combined score-ranked gene sets (hallmark, GSEA) from cluster 2 of the K-means clustering analysis comparing healthy kidneys, SARS-CoV-2 FISH-positive, SARS-CoV-2 FISH-negative, and non–COVID-19 ATI kidneys. (**C**) Bar plot of the top 10 normalized enriched score–ranked gene set (hallmark, GSEA) of FISH-positive COVID-19 kidneys compared with non–COVID-19 ATI kidneys (left panel). Hallmark IFN-α enrichment plot from GSEA comparing FISH-positive COVID-19 kidneys with non–COVID-19 ATI kidneys (right panel).

We next asked whether this molecular signature is specific to COVID-19 or is a common feature of kidney injury. Toward this aim, we compared the renal transcriptomic data of patients with COVID-19 with those of 6 patients with non–COVID-19 ATI to avoid bias because of morphological differences and to focus only on viral status in the kidney. PCA verified that gene expression was influenced by the presence of renal epithelial SARS-CoV-2 mRNA infection (Supplemental Figure 5). Moreover, K-means analysis showed a different pattern of gene expression in FISH-positive kidneys as compared with both FISH-negative and non–COVID-19 ATI kidneys. In particular, IFN-α and -γ response pathway (cluster 2) and cell–extracellular matrix interaction (cluster 3) were specifically enriched in FISH-positive kidneys (Figure 3, A and B). To further support the specific enrichment of IFN pathways in FISH-positive kidneys, we compared GSEA between FISH-positive kidneys and non–COVID-19 ATI kidneys, since the histological lesions were comparable between these groups of patients. Interestingly, GSEA identified the IFN-α pathway as the highest enriched gene set (NES: 1.85) in FISH-positive kidneys as compared with non–COVID-19 ATI kidneys (Figure 3C). Together these data suggest that the activation of the IFN-α pathway, a major event in innate antiviral immunity, may be used as a prognostic marker of direct renal viral infection.

**Figure 4:**
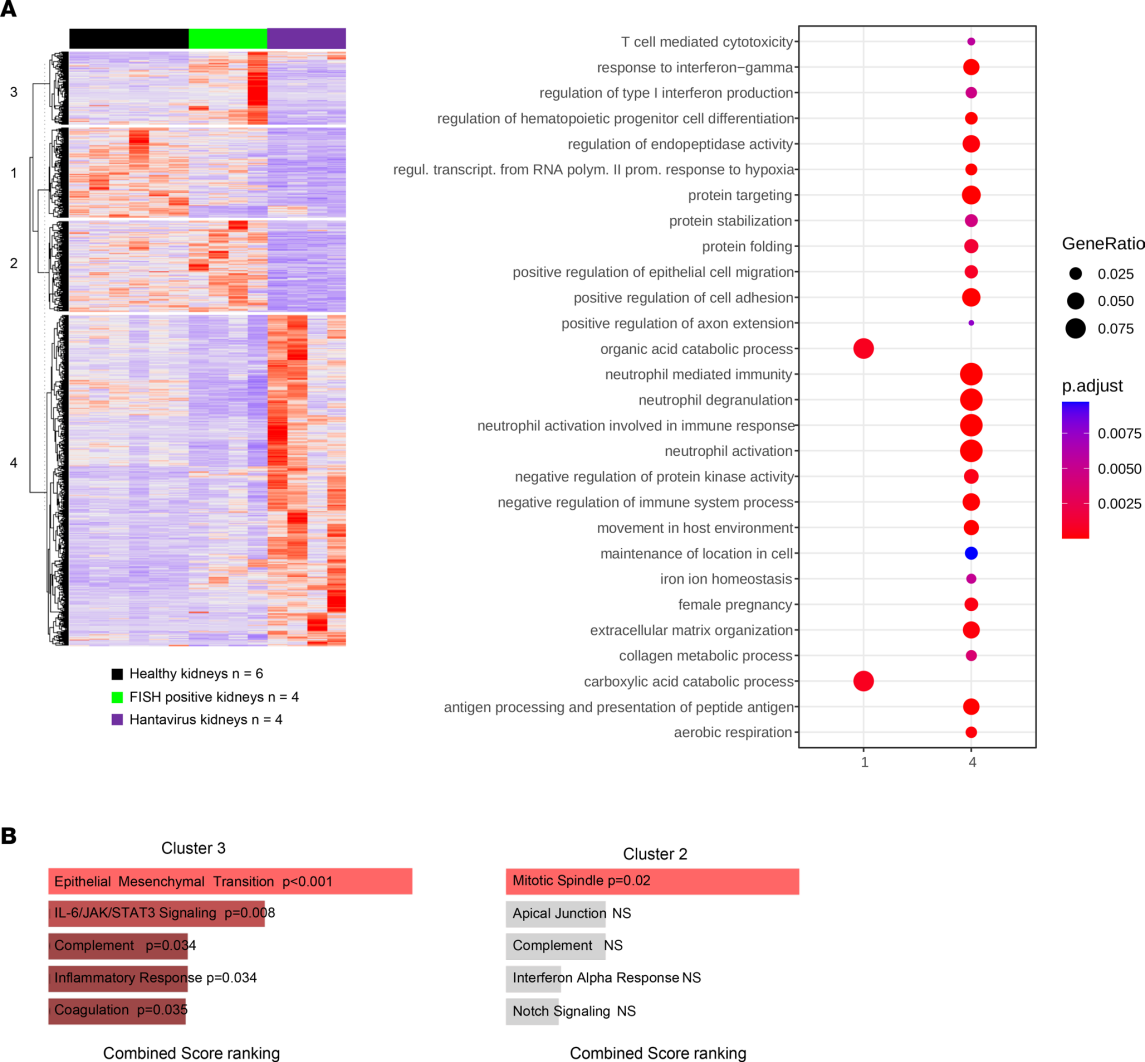
SARS-CoV-2 renal infection elicits a specific molecular signature compared with hantavirus kidneys. (**A**) Heatmap representing K-means analysis with the corresponding dot plot of the cluster profiling of DEGs comparing healthy kidneys and SARS-CoV-2 FISH-positive and hantavirus kidneys. (**B**) Bar plot of the top 5 combined score-ranked gene sets (hallmark, GSEA) from clusters 2 and 3 of the K-means clustering analysis comparing healthy kidneys and SARS-CoV-2 FISH-positive and hantavirus kidneys.

To verify whether these molecular signatures are specifically triggered by SARS-CoV-2 or are a general feature of virus kidney infection, we performed RNA-Seq on hantavirus-damaged kidney. The old-world hantavirus (*Orthohantavirus puumala*) is an RNA virus with renal tropism that leads to the same renal lesions as COVID-19, including ATI and microvascular inflammation (34). Our results showed that the renal transcriptome of these patients tends to segregate FISH-positive kidneys from hantavirus kidneys (Supplemental Figure 6). Moreover, K-means analysis revealed a particularly strong enrichment of immune response pathways in hantavirus kidneys compared with healthy kidneys (Figure 4A). In a similar way, fibrotic and proliferative pathways were particularly enriched in SARS-CoV-2–infected kidneys (Figure 4B). GSEA showed that several pathways, including oxidative phosphorylation, Myc targets, and protein secretion, were strongly induced in hantavirus compared with FISH-positive kidneys (Supplemental Figure 7). Finally, we compared only kidneys with the same lesion pattern, i.e., FISH-positive, hantavirus-damaged, and non–COVID-19 ATI kidneys. Interestingly, PCA uncovered that gene expression was significantly different between hantavirus and SARS-CoV-2–infected kidneys (Supplemental Figure 8). Consistently, K-means revealed specific immune pathway enrichment in hantavirus-damaged kidney, with an unexpected strong neutrophil activation signature (Supplemental Figure 9). Together, these findings reveal that the infection with different virus leads to specific signatures, despite a similar pattern of lesions.

### X-linked inhibitor of apoptosis-associated factor 1 is a critical target of renal SARS-CoV-2.

**Figure 5:**
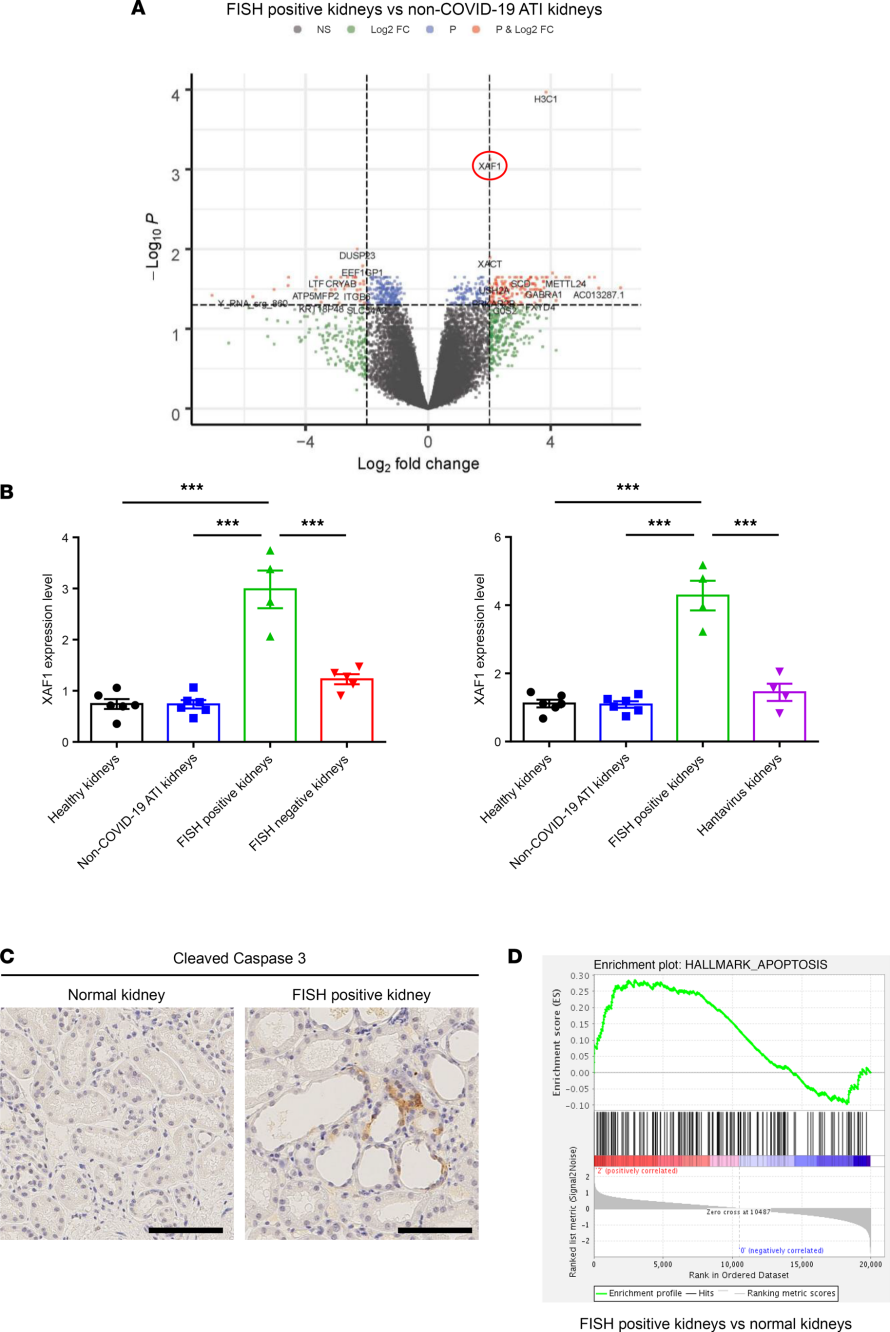
XAF1 is a critical target of renal SARS-CoV-2. (**A**) Volcano plot of DEGs comparing FISH-positive COVID-19 kidneys with non–COVID-19 ATI kidneys. (**B**) Comparative XAF1 gene expression in healthy kidneys, non–COVID-19 ATI kidneys, and FISH-positive and FISH-negative COVID-19 kidneys (left panel) and healthy kidneys, non–COVID-19 ATI kidneys, and FISH-positive and hantavirus kidneys (right panel). Tukey-Kramer test was applied to test the significance of the difference. ***P < 0.001. (**C**) Representative images of cleaved caspase-3 immunostaining in healthy kidneys and FISH-positive COVID-19 kidneys. (**D**) Hallmark apoptosis enrichment plot from GSEA comparing FISH-positive COVID-19 kidneys with healthy controls.

Finally, we performed a volcano plot analysis to identify the specific genetic targets of SARS-CoV-2 infection. A large number of genes were significantly up- or downregulated between COVID-19 FISH-positive patients and patients with non–COVID-19 ATI. Nevertheless, only 2 genes were highly specifically overexpressed in FISH-positive kidneys: H3C1 (H3 clustered histone 1) and X-linked inhibitor of apoptosis-associated factor 1 (XAF1, Figure 5A). Since XAF1 has been previously shown to be upregulated in inflammatory cells and epithelial lung cells infected with SARS-CoV-2 RNA (35, 36), we examined this target further. Quantitative mRNA expression level demonstrated that XAF1 was selectively upregulated in COVID-19 FISH-positive kidneys compared with non–COVID-19 ATI injured kidney and hantavirus-infected kidneys, supporting its specific upregulation in SARS-CoV-2–infected kidneys (Figure 5B). XAF1 is a tumor suppressor protein involved in cellular apoptosis, a cellular process known to participate in kidney damage during AKI. Consistently, cleaved caspase-3 staining revealed an increase in cell apoptosis in FISH-positive kidneys compared with healthy controls (Figure 5C). Moreover, GSEA showed an enrichment in apoptotic genes in FISH-positive kidneys compared with control kidneys (Figure 5D), supporting the potential role of apoptosis in SARS-CoV-2–induced renal lesions.

## Discussion

AKI is one of the most important complications in patients with COVID-19 and is considered a negative prognostic factor with respect to patient survival. Although a number of studies have investigated the pathophysiology of AKI during COVID-19, the majority of these studies were conducted on autopsies, raising the question of the relevance of these results for living renal cells. In this study, we used cutting-edge technologies to characterize the pathophysiology of kidney injury in a potentially unique large kidney biopsy series of living patients with SARS-CoV-2 infection collected at the time of kidney damage, including patients undergoing mechanical ventilation in an intensive care unit. The overarching aim was to capture the renal molecular signatures of COVID-19 and correlate them with SARS-CoV-2 infection. Our results verified that the major pathological feature of COVID-19 is ATI, which was associated with CG in patients with *APOL1* high-risk gene variants. We also identified a potentially novel pathological morphological entity characterized by the combination of thrombotic microangiopathy with C3 glomerulonephritis. Moreover, using appropriate controls, we clearly showed that SARS-CoV-2 infects living renal cells and that viral presence is associated with higher ACE2 protein expression. More importantly, transcriptomic analysis identified specific molecular signatures in SARS-CoV-2–positive kidneys, opening new therapeutic options to prevent one of the most severe complications of COVID-19.

ATI is the most frequent pathological lesion observed in patients with COVID-19–associated kidney disease. Our results indicate that these lesions were comparable to those observed in non–COVID-19–associated ATI, except for the presence of neutrophilic capillaritis. Neutrophils and neutrophil extracellular traps, 2 key actors of innate immunity, have emerged as a defining feature of severe COVID-19, at least in postmortem lung tissue (37, 38). Interestingly, we also observed a massive neutrophil infiltration in a pathological entity discovered here: TMA-C3GN. Even if TMA as well as C3GN have been previously reported in patients with COVID-19, this is the first time, to the best of our knowledge, that these 3 morphological lesions have been simultaneously observed in the kidney. It is worth noting that neutrophils’ and endothelial cells’ dysfunction, coagulation dysfunction, and complement activation have been reported to contribute to severe forms of COVID-19 (17, 19, 37).

Direct infection of SARS-CoV-2 in kidneys and its contribution to lesion development remains a controversial issue. Several technical limitations prevent any clear conclusion. For example, RT-PCR, one of the most used methods to detect SARS-CoV-2 in the kidney, cannot distinguish whether viral nucleic acids are present in the blood, urine, or inflammatory infiltrating or parenchymal cells (39). On the other hand, since the majority of the studies were conducted on kidneys from autopsies (39), it is not clear whether SARS-CoV-2 can directly infect living renal cells. More importantly, IHC does not seem appropriate to detect a specific SARS-CoV-2 nucleocapsid protein signal. Using appropriate controls, we clearly showed that the exact same immunoreactivity was observed in kidneys from COVID-19 and non–COVID-19 patients with ATI, suggesting that damaged renal tubules may express antigens that cross-react with anti SARS-CoV-2–specific antibodies. Similarly, May et al. failed to clearly detect SARS-CoV-2 nucleocapsid protein in a large series of 240 biopsies (27). In fact, although they could find a positive signal in 10 of 235 biopsies, SARS-CoV-2 mRNA experiments did not confirm this finding, suggesting a nonspecific staining. Together, these data raise concern about the specificity of SARS-CoV-2 antibodies. Using the robust technique of CoronaFISH, we provide what we believe is the first clear demonstration that SARS-CoV-2 infects renal cells. Owing to the use of 96 fluorophore-conjugated probes, CoronaFISH allows sensitive and specific visualization of the viral RNA in tissues at the single-cell level (40). Our study shows that SARS-CoV-2 targeted tubular cells. Since these cells are the most damaged in kidneys of severely affected COVID-19 patients, it is tempting to speculate that such infections may aggravate the extent of kidney damage. Consistently, the prevalence of severe ATI lesions and death was higher in FISH-positive patients compared with FISH-negative patients. In favor of this idea, it has been also observed that SARS-CoV-2 infection aggravates epithelial damage in the lungs of patients with COVID-19 (41).

ACE2, the receptor of SARS-CoV-2, is expressed in renal tubular cells (31–33). Remarkably, we observed that ACE2 expression was increased in SARS-CoV-2–positive kidneys as compared with SARS-CoV-2–negative kidneys, suggesting that higher receptor levels might favor viral infection. Conversely, other teams have shown that ACE2 expression decreases during coronavirus infection in lung (42, 43). If the increased expression of ACE2 in SARS-CoV-2–infected kidneys precedes tubular epithelial infection or is a direct or indirect consequence of it remains to be studied.

A major aim of our study was to define the molecular signatures of kidneys from patients with COVID-19. Compared with healthy controls, a significant number of genes related to inflammation, such as IFN-γ and -α responses and TNFA and IL-6 pathways, were highly enriched in COVID-19 kidneys. Although these molecular signatures have already been reported in the blood, lung, or airways (44, 45), our study provides the first evidence that these mechanisms play a role in renal deterioration upon COVID-19. Interestingly, we observed that the molecular signatures in SARS-CoV-2– and Hantavirus-damaged kidneys were significantly different despite a similar pattern of lesions. This further supports the idea that SARS-CoV-2 triggers a particular pattern of responses. Moreover, when we compared the expression profile of SARS-CoV-2–positive kidneys with non–COVID-19 ATI kidneys, we observed a strong activation of the IFN-α pathway. Anti-cytokines and anti-interferon therapies in COVID-19 critical illness have been extensively discussed (46). Our data suggest that the same therapies might be beneficial in protecting the kidney from SARS-CoV-2–induced lesions.

Another molecular signature that may deserve particular attention is the G2/M cell cycle checkpoint, which was strongly activated in SARS-CoV-2–positive kidneys as compared with non–COVID-19 ATI kidneys. Interestingly, a G2/M arrest has been shown to favor renal fibrosis after renal ischemia (47). Consistently, Jansen et al. have shown that patients with COVID-19 present with tubulointerstitial fibrosis and that SARS-CoV-2 infection stimulates profibrotic signaling in human kidney organoids (48). Whether this molecular signature predicts the development of chronic kidney disease in patients with SARS-CoV-2–positive kidneys is a risk factor that deserves to be monitored in long-term follow-up studies.

We also found the *XAF1* gene was specifically upregulated in SARS-CoV-2–positive kidney biopsies compared with controls, SARS-CoV-2–negative COVID-19 kidneys, and Hantavirus-infected kidneys. *XAF1* has been reported to be a highly and specific enriched gene in SARS-CoV-2–infected lung epithelial and immune cells (35, 36). *XAF1* is a tumor suppressor gene known to trigger apoptosis by counteracting the inhibitory effect of the IAP protein family that in turn inhibit caspases (49). Of interest, XAF1 has been shown to drive apoptosis of T cells in patients with COVID-19 (35). The observation that apoptosis was increased in SARS-CoV-2–infected kidneys and that apoptotic genes were enriched in the same tissue suggest a potential mechanistic role of XAF1 in renal tubular damage. Interestingly, apoptosis has been also shown to be increased in renal tubular epithelial cells in a mouse model (k18-hACE2 mice) of severe COVID-19 (50), reinforcing the possible role of programmed cell death in the pathophysiology of renal injury during SARS-CoV-2 infection. However, we cannot formally exclude that XAF1 is simply a biomarker of SARS-CoV-2 kidney infection. Further studies are required to demonstrate the possible mechanistic involvement of XAF1 in SARS-CoV-2–induced epithelial apoptosis.

We acknowledge that this work has 2 main limitations. First is the number of patients. However, we provide a likely unique cohort of living patients whose kidney samples were collected at the early time of SARS-CoV-2 infection, including patients with severe COVID-19 in intensive care units. Second is the quality of RNA. The degree of RNA degradation is inevitably related to this clinical context. In fact, the kidney biopsies were initially performed for a diagnostic purpose, and, therefore, kidney samples were not optimized for RNA preservation. To compensate for this limitation, we used specific tools adapted to the degraded RNA for the constitution of the RNA-Seq libraries (Ovation Universal RNA-Seq System) and performed loess correction in order to compensate for the difference in RNA integrity.

The relative lack of knowledge of the pathophysiology of kidney disease in patients with COVID-19 and associated controversial information have limited our ability to develop kidney-targeted therapeutic strategies able to treat and maybe prevent this severe life-threatening complication. By expanding the pathophysiological and molecular insights on the virus-kidney interplay in the setting of COVID-19, our study provides a solid background to design and test new candidate drugs to prevent renal failure in severely affected COVID-19 patients.

## Methods

### Study design and patients.

From March 2020 to March 2021, we prospectively enrolled 32 consecutive COVID-19 patients who underwent kidney biopsy 1–3 days from the onset of AKI and/or proteinuria in 7 French hospitals: Hôpital Européen Georges Pompidou (Paris, France), Centre Hospitalier Intercommunal de Poissy (Poissy, France), Centre Hospitalier Universitaire de la Guadeloupe (Pointe-à-Pitre, France), Hôpital Foch (Paris, France), Centre Hospitalier Universitaire de La Réunion (Saint Denis de La Réunion, France), Centre Hospitalier de Melun (Melun, France), and Hôpital Necker-Enfants Malades (Paris, France). Clinical and biological data available at kidney biopsy were collected. In the majority of cases, 2 cores of renal biopsies were obtained by percutaneous biopsies, one for light microscopy and the other for immunofluorescence and molecular studies. Lung autopsy material from 1 COVID-19 patient was provided by the human biological sample bank of the Lille COVID working group “LICORN” (Lille, France). Kidney and lung material from control patients originated from our Pathology Department at Hôpital Necker-Enfants Malades (Paris, France). In total, we studied as controls 6 patients with normal kidney histology (healthy controls), 6 patients with ATI prior to the COVID-19 pandemic, 4 patients with hantavirus-associated kidney lesions (Hôpital Necker-Enfants Malades, Paris, France, and CHU Lille, Lille, France), and 1 patient with normal lung histology. Healthy controls were individuals who were subjected to kidney biopsy because of a low-rate proteinuria (<1 g/L) or mild hematuria, with no microscopic abnormalities or immunofluorescent deposits. AThe TI control group was matched according to the degree of kidney ATI lesions. Demographic data of the healthy kidney control group are provided in Supplemental Table 1. Clinical, biological, and pathological findings of the hantavirus control group are provided in Supplemental Table 2.

### Histological analysis.

Kidney biopsies were fixed in formalin, alcohol, and acetic acid and paraffin-embedded. Four μm sections were stained with H&E, periodic acid–Schiff, Masson’s trichrome, and methenamine silver. Renal lesions were examined under a blinded protocol by 2 pathologists. Lesion quantifications were made according to the 2018 Banff Classification of Renal Allograft Pathology for interstitial inflammation, tubulitis, peritubular capillaritis, interstitial fibrosis, tubular atrophy, fibrous intimal thickening, and arteriolar hyalinosis (51). Interstitial edema and ATI, being a more often diffuse lesion process, was evaluated as absent being 0, mild being 1, or severe being 2.

### IHC and immunofluorescence.

For IHC, an automated IHC stainer BOND-III (Leica Biosystems) was used. Briefly, 4 μm sections of paraffin-embedded kidneys were submitted for appropriate antigen retrieval. Then, sections were incubated with the following antibodies: rabbit monoclonal anti-ACE2 antibody (Abcam, ab108252, 1:100), rabbit monoclonal anti–cleaved caspase-3 antibody (Cell Signaling Technology, 9664, 1:200), rabbit polyclonal anti–recombinant nucleoprotein from SARS-CoV antibody (a gift from Nicolas Escriou, Institut Pasteur, Paris, France), and rabbit polyclonal SARS-CoV-2 anti-nucleoprotein (Novusbio, NB100-56576, 1:500). ACE2 expression was evaluated by image quantification using the Integrated Density program of ImageJ software (NIH) on whole-kidney sections (×200). This measurement integrates the product of area and the mean intensity value above a specific threshold. This estimates the amount of the strength of the expression of a specific epitope.

Immunofluorescence was performed on frozen kidney biopsies using antibodies targeting the heavy chains of immunoglobulins (IgA, IgG, IgM), kappa and lambda light chains, complement (C3, C1q), and fibrinogen using the automated stainer BOND-III (Leica Biosystems). Immunofluorescence staining was performed on 26 of 32 biopsies.

### Vero cells and infection of cell lines.

Control and SARS-CoV-2–infected Vero cells originated from BetaCoV/France/IDF0372/2020. For IHC and FISH validation on FFPE samples, paraffin-embedded control and infected Vero cell suspensions were prepared using the Cytoblock Cell Block Preparation System (Thermo Fisher Scientific) according to the manufacturer’s instructions.

### RNA-FISH.

To visualize viral RNA molecules from SARS-CoV-2, we used the smFISH approach as previously described and validated (52). Briefly, 96 unlabeled primary probes were designed to specifically target the positive-stranded SARS-CoV-2 RNA and were prehybridized with fluorescently labeled secondary detector oligonucleotides for visualization (Cy5, 647). Images were acquired with a Spinning Disk Confocal Microscope (Yokogawa CSU-X1, Zeiss Axio-Observer Z1).

### APOL1 genotyping.

Six of the 10 patients with CG lesions based on kidney biopsy underwent *APOL1* genetic analysis using DNA extracted from peripheral blood. The search for SNPs defining the risk variants of the *APOL1* gene was done by real-time PCR genotyping with allelic discrimination (QuantStudio 7 Flex Real-Time PCR System; Applied Biosystems, Thermo Fisher Scientific). The probes were used to identify the following SNPs: s73885319 (p.S342G) and rs60910145 (p.I384M) for the G1 variant and the indel rs71785313 (p.NYK388K) for the G2 variant.

### RNA-Seq.

Transcriptomic analysis was performed on 21 native kidney biopsies: 12 control kidneys (6 normal healthy kidneys and 6 non–COVID-19 kidneys with ATI) and 9 COVID-19–associated kidney disease kidneys, of which 4 were FISH positive (SARS-CoV-2–infected kidneys) and 5 were FISH negative (uninfected kidneys). mRNA was extracted from nitrogen-frozen and OCT–embedded (CellPath) kidneys using the miRNeasy Kit (QIAGEN) according to the manufacturer’s instructions. The quantity and integrity of the purified RNA were assessed using the NanoDrop spectrophotometer ND-1000 (Thermo Fisher Scientific) and capillary electrophoresis (Agilent) for RNA integrity number (RIN).

Preparation of RNA sample libraries and RNA-Seq was performed by the Genomics Core Laboratory at Imagine Institute (Paris, France). Renal biopsies were originally frozen and embedded in OCT for immunofluorescence studies. This processing, which is not optimized for RNA preservation, inevitably led to a certain degree of RNA degradation. To compensate for this, the Ovation Universal RNA-Seq System (Nugen, Tecan) was used to prepare the RNA-Seq libraries. After a preliminary DNase digestion with a thermosensitive DNase (Arcticzyme), total RNA was reverse-transcribed, and a second strand of cDNA was synthesized. A fragmentation step was performed (or skipped for the most degraded total RNA samples) before Illumina-compatible indexed adaptor ligation. The ligation was followed by strand selection enzymatic reactions to keep the information about the orientation of the transcripts. Insert dependent adaptor cleavage reactions were performed to deplete all cDNA corresponding to human ribosomal transcripts before PCR enrichment. To ensure that no excessive amplification was performed during the final PCR step, the number of PCR cycles applied to each sample was evaluated in a preliminary quantitative PCR test using EvaGreen. An equimolar pool of final indexed RNA-Seq libraries was sequenced on the Illumina NovaSeq6000 (100 base paired-end reads), and about 50 million paired-end reads per library were produced.

### RNA-Seq analysis and statistics.

The quality of reads was assessed using FastQC and aligned to the GRCh38 human reference genome with HISAT2. Gene expression was quantified by htseq-count (version 0.13.5). Given the observed level of RNA degradation, we inspected the RNA integrity using RSeqC (https://sourceforge.net/projects/rseqc) (53). Only samples with a median OR and average transcript integrity number (TIN) > 30 were considered and subsequently adjusted using loess correction in order to compensate for the difference in RNA integrity (53). Among the 17 native COVID-19 kidney biopsies available for RNA extraction, 8 had fully degraded RNA (RIN < 1.5 and TIN < 30) and were not used for RNA-Seq analysis. The mean RIN and TIN of the 21 analyzed samples (COVID-19 and control) were, respectively, 6.6 ± 2.4 and 51 ± 6.7.

Differential expression was calculated with EdgeR, and lists of DEGs were generated by applying a 2-fold change cutoff and *P* value with Benjamini-Hochberg FDR multiple testings (*P* < 0.05) correction. Gene expression profile analyses (PCA and Venn diagram) were performed using R version 4.1.1. K-means gene expression clustering was performed using ComplexHeatmap_2.8.0 on R. Cluster profiling from K-means was performed using clusterProfiler_4.0.5 on R. When no cluster profiles were identified using clusterProfiler_4.0.5, cluster profiling was performed with Enrichr (https://maayanlab.cloud/Enrichr/) using GSEA hallmark gene set.

As the RNA-Seq analysis of the hantavirus control group was performed later in another batch, to allow accurate differential expression evaluations between groups, we have re-sequenced the healthy, non–COVID-19 ATI and FISH-positive kidney groups and performed batch effect adjustment for RNA-Seq count data using ComBat-seq (54).

GSEA (version 4.1.0) was used to evaluate the transcriptional profiles between groups as previously described (https://www.gsea-msigdb.org/gsea/index.jsp).

RNA-Seq data for all samples have been deposited in the NCBI Gene Expression Omnibus (accession number GSE202182).

### Statistics.

Clinical and biological data are expressed as mean (quantitative variables) or ratio (categorical variables). Differences between groups were evaluated using either 1-way ANOVA followed by, when significant (*P* < 0.05), the Tukey-Kramer test for quantitative variables or Fisher’s exact test for qualitative variables. Statistical analyses were performed using GraphPad Prism.

### Study approval.

The protocol was approved by the Institutional Review Board of Hôpital Necker-Enfants Malades, and informed written consent was obtained from all patients.

## Author contributions

PI and MR contributed to conception of the study and to acquisition, analysis, and interpretation of the data and wrote the manuscript; PV, MJ, ME, SG, VG, AJE, CG, FP, MZ, ER, SP, and JBG contributed to acquisition and analysis of the data and editing of the manuscript; AK, JPDVH, TJM, FM, CZ, and GBS contributed to analysis and interpretation of the data and editing of the manuscript; MP and FT contributed to conception of the study and analysis and interpretation of the data and wrote the manuscript; and PI, MR, MJ, VG, AJE, CG, FP, AK, DA, and JPDVH took care of the reported patients. All the authors facilitated the study and declare they have seen and approved the final version of the manuscript.

## Supplementary Material

Supplemental data

## Figures and Tables

**Table 1:**
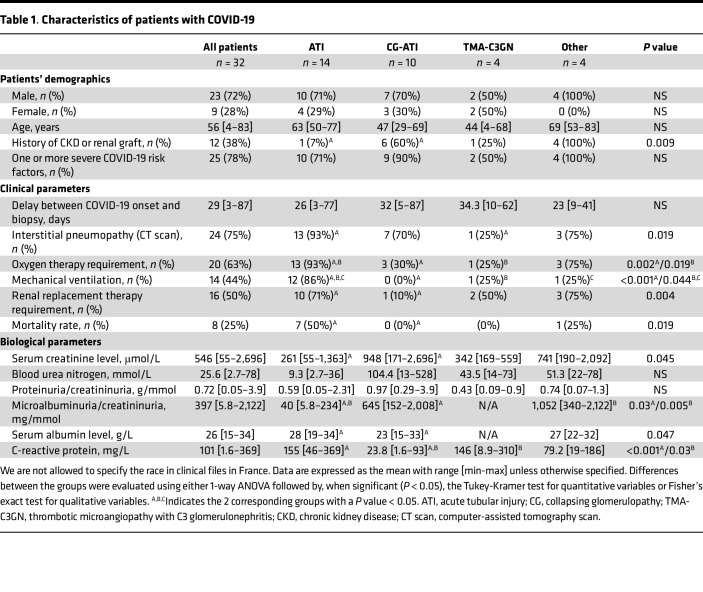
Characteristics of patients with COVID-19

## Version 1 02/07/2023

In-Press Preview

## Version 2 03/08/2023

Electronic publication
