# Supplementary material for: A specific molecular signature in SARS-CoV-2–infected kidney biopsies
Source: JCI Insight. 2023 Mar 8;8(5):e165192. doi: 10.1172/jci.insight.165192 (PMC10077488; doi:10.1172/jci.insight.165192)
Supplement: Supplemental data [file jciinsight-8-165192-s115.pdf]

Table S1. Clinical data of patients with normal (healthy) kidney biopsy

Patient	Sex	Age
**HK 1**	M	17
**HK 2**	M	30
**HK 3**	M	58
**HK 4**	F	30
**HK 5**	F	40
**HK 6**	M	35

Table S2. Clinical and biological data of patients with Hantavirus kidney biopsy

Patient	Sex	Biological Parameters	Age	Glomerulosclerosis (Fraction)	IFTA	I	PTC	Oedema	ATI	Interstitial hemorrhage
**Hantavirus #1**	M	AKI	23	0/20	0	+	++	1	2	0
**Hantavirus #2**	F	AKI<br>Proteinuria 3.3g/g,<br>Hematuria	23	0/12	0	0	+	2	2	++
**Hantavirus #3**	M	AKI<br>Proteinuria >5g/g<br>No hematuria	35	0/18	0	++	++	2	2	0
**Hantavirus #4**	M	AKI<br>Proteinuria<br>No hematuria	35	0/16	0	0	0	2	2	0

AKI: acute kidney injury; IFTA: interstitial fibrosis and tubular atrophy; I: Interstitial Inflammation; PTC: Peritubular Capillaritis; ATI: acute tubular injury.

Table S3. Individual clinical and biological characteristics of COVID-19 patients

Patient's demographic						Clinical Parameters					Biological Parameters					
ID	Kidney lesions	Age	Sex	History of CKD (KDIGO)	History of severe COVID-19 risk factors	Dg Tool	MV	KB delay (day)	RRT	Death	sCr µmol/l	eGFR ml/min	sAlb g/l	Pu g/mmol	Albu mg/mmol	CRP mg/ml
1*	ATI	65	M	no	HBP	PCR	yes	19	yes	no	114	59	27	0.10	12	225
2*	ATI	71	M	no	Smoking, HBP, Obesity	PCR	yes	8	yes	**yes**	76	113	N/A	0.08	8.4	221
3	ATI	50	F	no	None	PCR	yes	3	yes	**yes**	78	98	N/A	0.12	35	N/A
4	ATI	59	F	no	HBP	PCR	yes	40	no	**yes**	182	26	34	0.05	6.2	75.4
5	ATI	58	M	no	None	PCR	yes	12	yes	no	55	141	27	0.16	22	155
6	ATI	65	F	I	Renal Graft, Diabetes, HBP, CVD, IS	PCR	yes	59	yes	no	280	19	27.2	2.31	N/A	155.7
7	ATI	72	M	no	None	PCR	yes	17	no	**yes**	57	116	N/A	0.20	10	281
8*	ATI	63	M	no	IS	PCR	no	54	no	no	624	8	19	1.49	N/A	46
9	ATI	73	M	no	HBP, Cancer	PCR	yes	17	yes	**yes**	99	68	29	N/A	N/A	369
10	ATI	71	M	no	None	PCR	yes	18	no	**yes**	97	70	N/A	0.09	N/A	N/A
11	ATI	50	M	no	Obesity	PCR	yes	10	yes	no	82	92	28	0.75	5.8	155
12	ATI	58	M	no	None	PCR	yes	18	yes	**yes**	94	85	32	0.13	26	72
13	ATI	77	M	no	HBP	PCR	no	16	yes	no	1363	3	30	0.10	N/A	85
14*	ATI	55	F	no	Diabetes, HBP, IS	PCR	yes	77	yes	no	454	11	29	2.10	234	58
15	CG-ATI	29	M	III	Renal Graft, Asthma, HBP, IS	PCR	no	5	no	no	534	14	33	0.81	490	93
16	CG-ATI	44	M	no	Diabetes	Serology	no	87	no	no	671	9	24	0.50	N/A	1.9
17*	CG-ATI	69	F	II	HBP	PCR	no	10	no	no	171	25	17	0.80	N/A	4.6
18	CG-ATI	67	F	no	None	PCR	no	25	no	no	468	10	N/A	0.33	N/A	21.8
19	CG-ATI	30	F	no	HBP, Obesity	PCR	no	33	no	no	672	9	22	0.39	231.2	11
20*	CG-ATI	33	M	V	HBP, CVD	Serology	no	73	yes	no	2086	3	20	3.90	2008	5
21*	CG-ATI	34	M	IV	HBP	PCR	no	22	no	no	961	7	15	0.75	N/A	1.6
22	CG-ATI	48	M	no	Diabetes, HBP, Obesity	PCR	no	26	no	no	2696	2	27	1.33	N/A	4.4
23	CG-ATI	66	M	II	None	PCR	no	20	no	no	691	7	30	0.29	152	89
24	CG-ATI	48	M	III	HBP, CVD, IS	PCR	no	15	no	no	528	10	22	0.58	344.8	6
25	TMA/C3GN	46	M	no	HBP, Obesity	Serology	no	N/A	yes	no	169	40	N/A	0.90	N/A	310
26	TMA/C3GN	4	F	no	None	PCR	no	10	yes	no	380	7	N/A	N/A	N/A	8.9
27	TMA/C3GN	68	F	no	None	PCR	no	31	no	no	559	7	N/A	0.09	N/A	180
28	TMA/C3GN	56	M	III	Renal Graft, HBP, CVD, IS	PCR	yes	62	no	no	260	23	N/A	0.30	N/A	85.5
29	Other	76	M	III	Renal Graft, Diabetes, IS, HBP, CVD	PCR	yes	9	yes	**yes**	190	32	32	0.07	340	186
30*	Other	83	M	II	Diabetes, HBP, CVD	PCR	no	14	no	no	238	25	28	0.39	2122	19
31	Other	64	M	III	Diabetes, HBP, Obesity	PCR	no	41	yes	no	2092	3	N/A	1.20	N/A	52.7
32*	Other	53	M	III	Smoking, HBP, IS	PCR	no	29	yes	no	444	22	22	1.30	695	59

CKD : Chronic Kidney Disease; KDIGO : Kidney Disease Improvement Global Outcomes; Dg: Diagnosis; MV: Mechanical Ventilation; KB : Kidney biopsy; RRT :Renal Replacement Therapy; sCr : Serum Creatinine; eGFR : estimated Glomerular Filtration Rate (MDRD); sAlb : Serum Albumine; Pu : Proteinuria; Albu : Albuminuria; CRP : C Reactive Protein; ATI : Acute Tubular Injury; HBP : High Blood Pressure; PCR : Polymerase Chain Reaction; CVD : Cardiovascular Disease; IS : Immunosuppressive treatment; CT-scan : Computerized Tomography scanner; CG: Collapsing Glomerulopathy; TMA/C3GN: Thrombotic Microangiopathy/C3 Glomerulonephritis. N/A: Not Available.

** Indicates patients with RNA-seq analysis*

Table S4. Individual histological characteristics of 32 COVID-19 patients and 6 non-COVID-19 ATI patients

ID	Kidney lesions	Number of Glomeruli/sclerotic	FSGS	TMA	Other	ATI	Edema	I / i-IFTA	T	IFTA	PTC	CV / AH	Immunofluorescence	FISH
1*	**ATI**	10/1	no	no	no	1	1	0 / 0	0	0	1 (PMN)	2 / 1	-	+
2*	**ATI**	37/6	no	no	Ischemic	2	1	0 / 0	0	1	0	3 / 1	N/A	+
3	**ATI**	30/4	no	no	no	2	2	0 / 0	0	1	0	1 / 0	-	-
4	**ATI**	27/1	no	no	Ischemic	2	0	0 / 0	0	1	1 (PMN)	1 / 2	-	+
5	**ATI**	15/0	no	no	no	1	0	0 / 0	0	0	0	1 / 2	-	-
6	**ATI**	12/3	no	no	no	1	1	0 / 0	0	0	2	2 / 1	N/A	+
7	**ATI**	26/2	no	no	no	1	1	0 / 0	0	0	0	2 / 1	-	+
8*	**ATI**	0/0	NA	no	no	2	2	0 / 0	0	2	1	N/A / 0	N/A	+
9	**ATI**	26/7	no	no	no	1	2	0 / 1	0	1	1	2 / 2	-	+
10	**ATI**	9/1	no	no	no	1	1	0 / 0	0	0	0	3 / 2	-	+
11	**ATI**	35/5	no	no	Ischemic	1	1	0 / 0	0	0	1 (PMN)	2 / 2	N/A	+
12	**ATI**	23/0	no	no	no	2	2	0 / 0	0	0	2 (PMN)	2 / 2	-	+
13	**ATI**	13/3	no	no	no	2	2	1 / 2	0	1	0	1 / 2	N/A	+
14*	**ATI**	10/2	no	no	no	2	1	0 / 2	3	3	1	3 / 3	-	-
15	**CG-ATI**	5/1	Collapsing	no	no	1	0	0 / 0	1	1	0	2 / 2	N/A	-
16	**CG-ATI**	15/2	Collapsing	no	no	1	2	0 / 1	1	2	0	2 / 1	-	N/A
17*	**CG-ATI**	5/0	Collapsing	no	no	2	2	0 / 0	0	1	0	2 / 0	-	+
18	**CG-ATI**	7/2	Collapsing	no	no	2	2	0 / 1	1	2	3	2 / 0	N/A	+
19	**CG-ATI**	34/8	Collapsing	no	no	1	0	0 / 1	2	0	0	1 / 2	-	+
20*	**CG-ATI**	12/6	Collapsing	no	no	2	1	0 / 1	1	3	0	2 / 3	-	-
21*	**CG-ATI**	17/10	Collapsing	no	no	2	1	0 / 1	1	3	1 (PMN)	2 / 2	-	-
22	**CG-ATI**	2/0	Collapsing	no	no	2	2	0 / 1	3	2	1 (PMN)	N/A /1	-	+
23	**CG-ATI**	15/2	Collapsing	yes	no	2	0	1 / 2	2	2	1 (PMN)	3 / 2	-	-
24	**CG-ATI**	11/5	Collapsing	no	no	2	1	0 / 2	2	1	1	3 / 1	Mesangial C3	+
25	**TMA/C3GN**	20/3	no	**yes**	no	2	2	1 / 2	3	1	0	2 / 2	Mesangial C3	-
26	**TMA/C3GN**	62/0	no	**yes**	Prolif GN	1	0	0 / 0	0	0	0	0 / 0	Parietal IgG/C3	-
27	**TMA/C3GN**	16/3	no	**yes**	Prolif GN	1	2	0 / 0	0	0	0	0 / 2	Mesangial C3	-
28	**TMA/GN**	11/5	NOS	**yes**	Prolif GN	1	1	0 / 1	3	2	2	2 / 2	N/A	-
29	**DN**	7/1	NOS	no	DN	1	0	0 / 0	0	3	0	3 / 2	N/A	N/A
30*	**DN**	9/6	NOS	no	DN	1	0	0 / 1	1	3	0	N/A / 3	-	-
31	**ATI, FSGS**	5/3	NOS	no	no	2	1	0 / 1	0	3	0	1 / 2	-	-
32*	**FSGS**	10/5	NOS	no	no	3	1	0 / 3	3	1	0	1 / 2	-	-
#1*	**ATI**	31/0	NOS	no	no	2	2	0 / 0	0	0	0	3 / 2	-	0
#2*	**ATI**	16/6	no	no	Ischemic	2	0	0 / 0	0	1	2	1 / 2	Mesangial IgA/C3	0
#3*	**ATI**	20/2	no	no	Ischemic	1	1	0 / 1	0	1	0	2 / 2	-	N/A
#4*	**ATI**	14/0	no	no	no	2	1	0 / 0	0	0	0	0 / 2	-	N/A
#5*	**ATI**	16/0	no	no	no	1	0	0 / 0	0	1	2	2 / 0	-	N/A
#6*	**ATI**	18/2	no	no	no	2	0	0 / 0	0	1	0	1 / 2	-	N/A

*FSGS: Focal Segmental Glomerulosclerosis; TMA: Thrombotic Microangiopathy; ATI: Acute Tubular Injury; I: Interstitial Inflammation; i-IFTA: Inflammation in fibrotic areas; T: Tubulitis; IFTA: Interstitial Fibrosis and Tubular Atrophy; PTC: Peritubular Capillaritis; CV: Fibrous Intimal Thickening; AH: Arteriolar Hyalinosis; FISH: Fluorescent In Situ Hybrization; PMN: Polymorphonuclear Neutrophils; CG: Collapsing Glomerulopathy; TMA/C3GN: Thrombotic Microangiopathy/C3 Glomerulonephritis; NOS: Not Otherwise Specified; Prolif GN: Proliferative Glomerulonephritis; DN: Diabetic Nephropathy; IgA: Immunoglobulin A. N/A: Not Available. * Indicates patients with RNA-seq analysis*

Table S5. Clinical and pathological characteristics of COVID-19 ATI and non-COVI-19 ATI

	COVID-19 ATI n = 14	Non-COVID-19 ATI n = 6	P Value
**Patients' demographic**			
Age; years	63 [50-77]	61 [35-85]	0.68
Male; n (%)	10 (71%)	3 (50%)	0.61
**Biological parameters**			
Serum creatinine level; µmol/L	261 [55-1363]	413 [247-633]	0.39
Proteinuria/creatininuria; g/mmol	0.59 [0.05-2.31]	0.4 [0.26-0.8]	0.66
**Glomerular characteristics**			
Number of total glomeruli	19.5 [0-37]	19.5 [14-31]	0.95
Number of sclerotic glomeruli, n (%)	35 (13%)	10 (9%)	0.30
**Tubulo-interstitial characteristics**			
ATI	1.5 [1-2]	1.6 [1-2]	0.51
Interstitial edema,	1.2 [0-2]	0.6 [0-2]	0.14
Interstitial inflammation	0.07 [0-1]	0 [0-0]	0.53
i-IFTA	0.4 [0-2]	0.2 [0-1]	0.57
Tubulitis	0.2 [0-3]	0 [0-0]	0.53
IFTA	0.6 [0-3]	0.7 [0-1]	0.95
**Vascular characteristics**			
Peritubular capillaritis (PTC)	0.7 [0-2]	0.7 [0-2]	0.82
Neutrophilic PTC; n (%)	4 (29%)	0 (0%)	0.27
Fibrous intimal thickening	1.9 [1-3]	1.5 [0-3]	0.33
Arteriolar hyalinosis	1.5 [0-3]	1.7 [0-2]	0.69

ATI: Acute Tubular Injury; i-IFTA: Inflammation in fibrotic areas; IFTA: Interstitial Fibrosis and Tubular Atrophy. Data are expressed as mean with range [Min-Max], useless otherwise specified. Differences between the groups were evaluated using either one-way ANOVA followed by, when significant ($p< 0.05$), the Tukey-Kramer for quantitative variable or Fisher's exact test for qualitative variable. P value < 0.05 is considered significant.

Table S6. Clinical and biological data of patients with C3GN and TMA lesions

Patient	Age	Sex	FISH	Biological TMA	Hb (g/dL)	Platelets count ($10^3/mm^3$)	LDH (UI/L)	Haptoglobin g/L	Schistocytes	Complement biology	Complement genetic
**Patient 25**	46	M	negative	no	6.2	90	636	2.91	no	Normal C3<br>Low C4	normal
**Patient 26**	4	F	negative	no	6.8	182	565	1.50	NA	Low C3<br>Normal C4	NA
**Patient 27**	68	M	negative	no	9	137	210	NA	no	Low C3<br>Low C4	NA
**Patient 28**	56	F	negative	no	9.3	234	378	1.61	no	Normal C3<br>Normal C4	NA

NA: non available; Complement genes analyzed: CFH, CFI, CD46, C3, CFB and THBD

Table S7. Histological data of patients with C3GN and TMA lesions.

Patient	Glomerular Thrombi	Arteriolar Thrombi	Mesangiolysis	Fractured RBC	Arteriolar intimal oedema	Endocapillary hypercellularity	Crescents	IF findings
**Patient 25**	yes	yes	no	no	yes	no	no	C3
**Patient 26**	yes	yes	no	no	yes	yes	no	C3+++ IgG+
**Patient 27**	yes	yes	yes	no	yes	yes	no	C3
**Patient 28**	yes	no	yes	no	no	yes	no	NA

Table S8. Clinical, biological and pathological characteristics of FISH positive and FISH negative patients

	FISH positive (n=16)	FISH negative (n=14)	P Value
**Age**, years, mean (min-max)	61.6 (30-77)	49.9 (4-83)	**0.05**
**Sex**, male, n (%)	11 (68.8)	10 (71.4)	1.0
**History of CKD**, n (%)	3 (18.8)	8 (57.1)	0.06
**Delay of kidney biopsy**, days, mean (min-max)	24.1 (8-59)	30.7 (3-77)	0.67
**Renal replacement therapy**, n (%)	7 (43.8)	8 (57.1)	0.72
**Death**, n (%)	8 (50)	1 (7.1)	**0.02**
**Serum creatinine**, µmol/L, mean (min-max)	475 (57-2696)	643 (55-2092)	0.27
**eGFR**, mL/min, mean (min-max)	44.7 (2-116)	29.1 (3-141)	0.24
**Serum albumine**, g/L, mean (min-max)	26.1 (17-34)	25.5 (15-33)	0.92
**CRP**, mg/mL, mean (min-max)	147 (6-369)	108 (2-310)	0.69
**Proteinuria**, g/g creatininuria, mean (min-max)	0.6 (0.1-2.3)	0.9 (0.1-3.9)	0.24
**Histological parameters**			
ATI, n (%)	11 (68.8)	3 (21.4)	**0.002**
Collapsing/ATI, n (%)	5 (31.3)	4 (28.6)	1.0
TMA/C3GN, n (%)	0	4 (28.6)	**0.04**
Other, n (%)	0	3 (21.4)	**0.09**
Glomerulosclerosis, % (min-max)	13.5 (0-33)	30.7 (0-67)	**0.01**
Interstitial Fibrosis (IFTA), mean (min-max)	0.75 (0-3)	1.64 (0-2)	**0.02**

CKD: Chronic Kidney Disease; eGFR: estimated Glomerular Filtration Rate (MDRD); CRP: C Reactive Protein; ATI: Acute Tubular Injury; TMA/C3GN: Thrombotic Microangiopathy/C3 Glomerulonephritis

SUPPLEMENTARY FIGURE LEGEND

Figure S1: Clinical characteristics and renal morphological findings. A: Heat map representing the clinical and biological data of the 32 COVID-19 patients. Categorical variables are represented for each patient, with red indicating their presence and white their absence. Quantitative variables are represented for each patient, with 4 levels of red intensity corresponding to the 4 quartiles of the variable. Grey square indicates "not available" data. HBP: High Blood Pressure; CVD: Cardio Vascular Disease; MV: Mechanical Ventilation; RRT: Renal Replacement Therapy; sCr: serum Creatinine; sAlb: serum Albumin; AlbU: Albuminuria: CRP: C Reactive Protein; FISH: Fluorescent in Situ hybridization. **B**: Kidney morphology of COVID-19 patients with acute tubular injury (ATI) lesions. Masson Trichrome staining shows dilated tubular lumens, flattened epithelia and the presence of cellular debris and hyaline cast in tubules (red arrows). The right image shows peritubular capillaritis composed of polynuclear cells (black square). **C**: Kidney morphology of COVID-19 patients with Collapsing Glomerulopathy and ATI lesions. Masson Trichrome staining (left) and silver staining (right) shows global glomerular tuft collapse with overlying podocyte hyperplasia and hypertrophy and numerous protein droplets within the podocyte cytoplasm. **D**: Kidney morphology of COVID-19 patients with Thrombotic Microangiopathy and C3-Glomerulonephritis. Upper panels: Masson Trichrome staining showing glomerular (left) and arteriolar (right) fibrinous thrombosis (red arrow). Lower panels: periodic acid–Schiff staining (left) shows global endocapillary proliferation composed of numerous neutrophils and immunofluorescence (right) shows glomerular complement C3 deposits. Scale bars in all panels: 100 µm.

Figure S2: Immunohistochemical detection of SARS-CoV-2 nucleocapsid protein. A: Results of SARS-CoV-2 immunostaining using an antibody directed against the recombinant nucleocapsid protein on uninfected and SARS-CoV-2 paraffin-embedded infected Vero cells

and lung tissue from healthy and COVID-19 patients. **B:** SARS-CoV-2 immunostaining using the same recombinant anti-N antibody on kidneys from healthy individuals, non-COVID-19 ATI patients and COVID-19 patients. **C**: SARS-CoV-2 immunostaining using a commercial (Novusbio) anti-N antibody on uninfected and SARS-CoV-2 paraffin-embedded infected Vero cells and kidneys from non-COVID-19 ATI patients.

Patients with non-COVID-19 ATI displayed numerous false positive tubular sections (red arrows). Scale bars: 100 μm.

Figure S3: **ACE-2 expression levels correlate with renal SARS-CoV-2 infection**. **A**: Results of ACE2 immunostaining on healthy kidney showing cortical expression of ACE2 within tubular cells. **B**: Results of ACE2 immunostaining (left panel) and quantification (right panel) in kidney tissues from FISH positive and FISH negative COVID-19 patients. Student T test was applied to test the significance of the difference. * $P < 0.05$. Scale bars: 100 μm.

Figure S4: RNAseq reveals differentially expressed genes in kidneys from healthy, COVID-19, non-COVID-19 ATI and Hantavirus patients. Number of differentially expressed genes (total, up, down and) comparing: COVID-19 kidney Vs Healthy kidney; FISH positive kidney Vs Healthy kidney; FISH negative kidney Vs Healthy kidney; ATI kidney Vs Healthy kidney; FISH positive Kidney Vs ATI kidney; FISH negative Kidney Vs ATI kidney FISH positive Kidney Vs FISH negative Kidney; Hantavirus Vs Healthy kidney, Hantavirus vs FISH positive kidney.

Figure S5: Principal component analysis with non-COVID-19 ATI kidneys. Principal component analysis of the top 500 most variable genes in COVID-19 FISH positive and FISH negative, non-COVID-19 ATI and healthy kidneys.

Figure S6: Principal component analysis with Hantavirus kidneys. Principal component analysis of the top 500 most variable genes in all patients from in COVID-19 FISH positive, Hantavirus and healthy kidneys.

Figure S7: GSEA analysis of Hantavirus compared to healthy and COVID-19 FISH-positive kidneys. Bar plot of the top 25 normalized enriched score-ranked gene sets (Hallmark, GSEA) with $q < 0.05$ of Hantavirus kidneys compared to either healthy (upper panel) or COVID-19 FISH-positive kidneys (lower panel).

Figure S8: Principal component analysis with Hantavirus and Non-COVID-19 ATI kidneys. Principal component analysis of the top 500 most variable genes in COVID-19 FISH positive, Hantavirus and non-COVID-19 ATI kidneys.

Figure S9: Gene expression profiling comparing FISH positive, Hantavirus and Non-COVID-19 ATI kidneys. A: Heat map representing K-means analysis with the corresponding dot plot of the cluster 1 profiling of differentially expressed genes comparing COVID-19 FISH-positive, Hantavirus and non-COVID-19 ATI kidneys. **B:** Bar plot of the top ten combined score-ranked gene set (Hallmark, GSEA) from cluster 5 (upper panel) and 2 (lower panel) of the K-means clustering analysis comparing FISH-positive, Hantavirus and non-COVID-19 ATI kidneys.

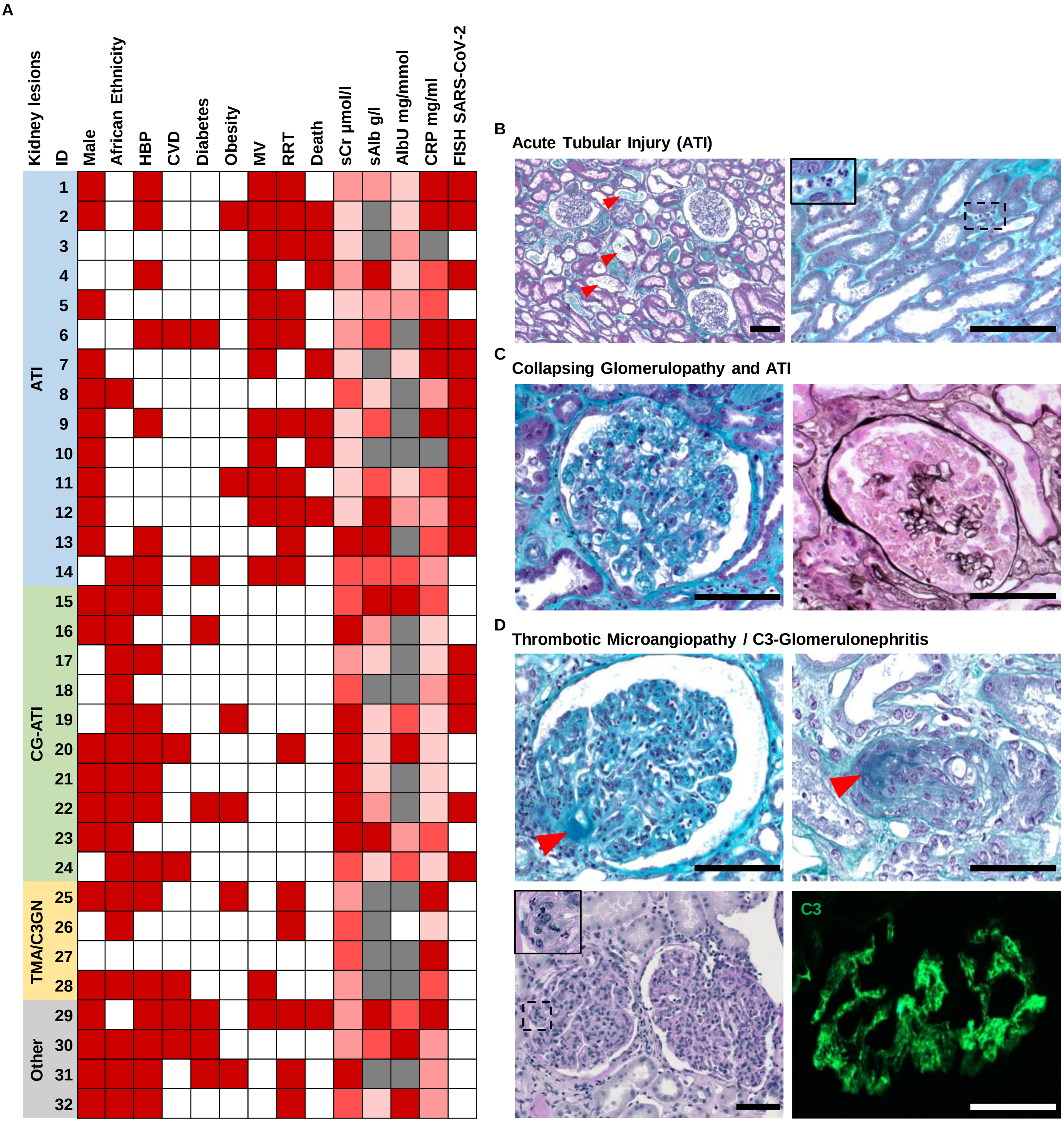
A
Kidney lesions
ID
Male
African Ethnicity
HBP
CVD
Diabetes
Obesity
MV
RRT
Death
sCr µmol/l
sAlb g/l
AlbU mg/mmol
CRP mg/ml
FISH SARS-CoV-2
ATI
CG-ATI
TMA/C3GN
Other
1
2
3
4
5
6
7
8
9
10
11
12
13
14
15
16
17
18
19
20
21
22
23
24
25
26
27
28
29
30
31
32
B
Acute Tubular Injury (ATI)
C
Collapsing Glomerulopathy and ATI
D
Thrombotic Microangiopathy / C3-Glomerulonephritis
C3

Figure S1

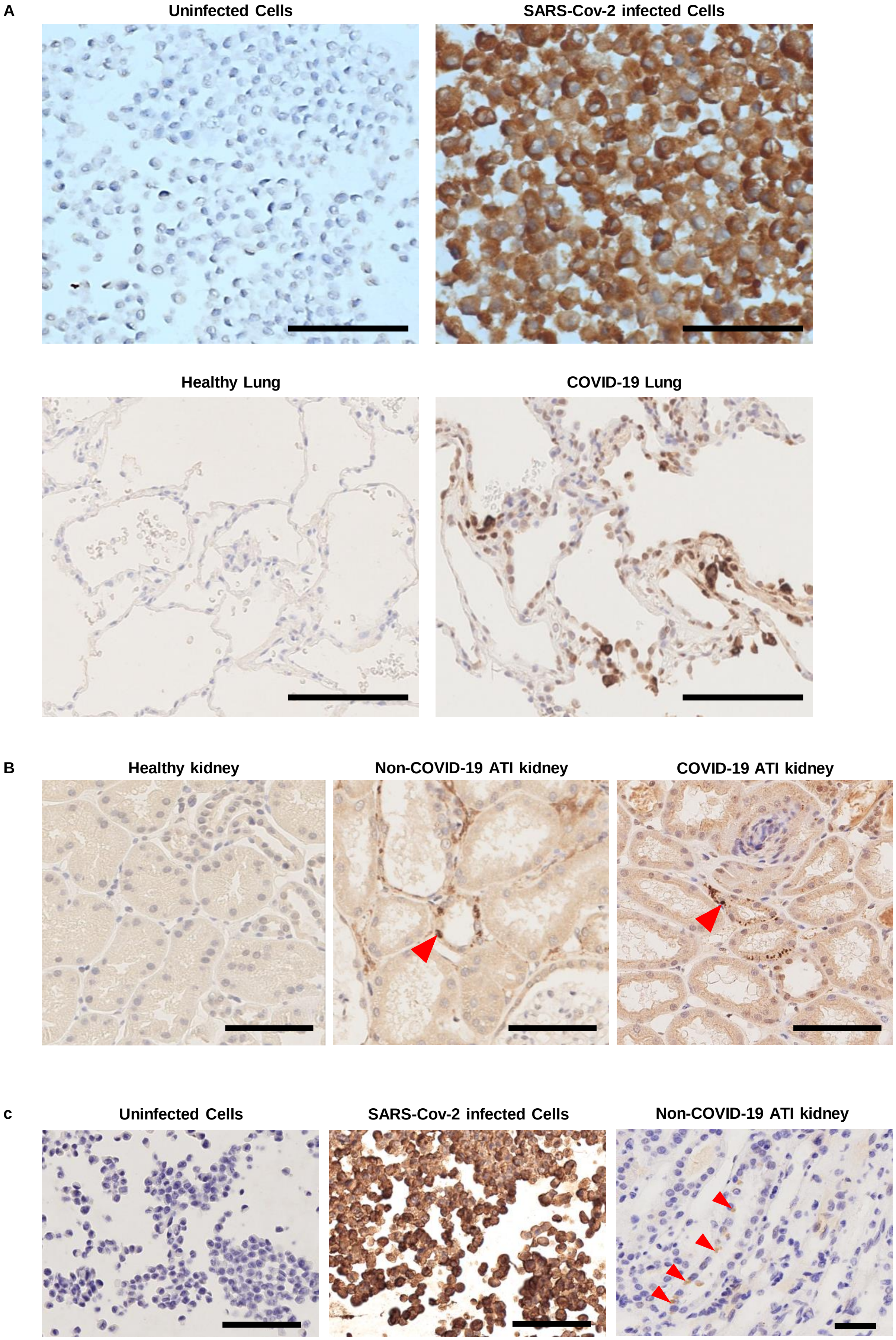
A
Uninfected Cells
SARS-Cov-2 infected Cells
Healthy Lung
COVID-19 Lung
B
Healthy kidney
Non-COVID-19 ATI kidney
COVID-19 ATI kidney
c
Uninfected Cells
SARS-Cov-2 infected Cells
Non-COVID-19 ATI kidney

Figure S2

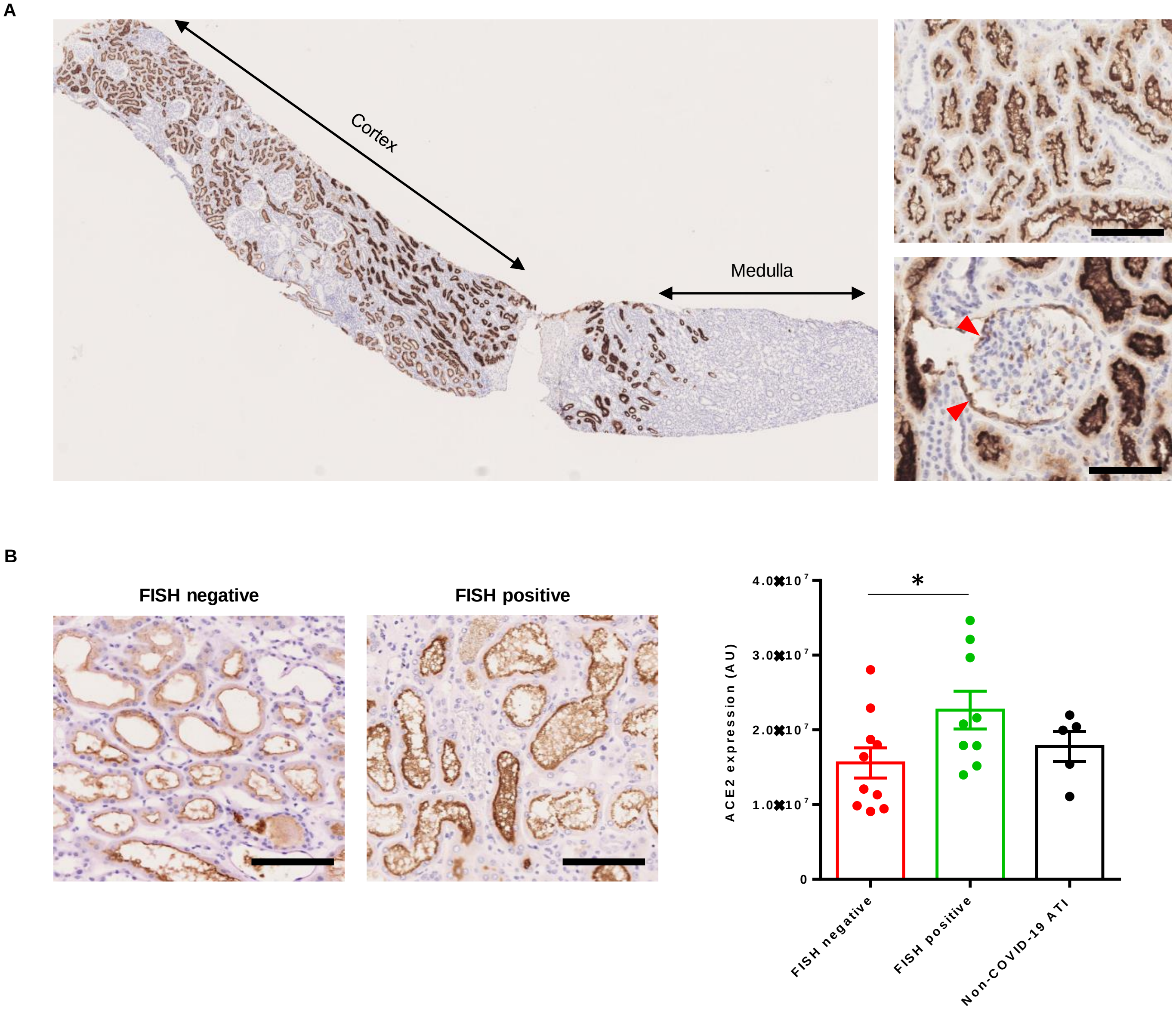
A
Cortex
Medulla
B
FISH negative
FISH positive
4.0×10⁷
3.0×10⁷
2.0×10⁷
1.0×10⁷
0
ACE2 expression (AU)
*
FISH negative
FISH positive
Non-COVID-19 ATI

Figure S3

COVID-19 kidney Vs Healthy kidney	Total	785
	Up	324
	Down	461
FISH positive kidney Vs Healthy kidney	Total	658
	Up	381
	Down	277
FISH negative kidney Vs Healthy kidney	Total	885
	Up	439
	Down	446
ATI kidney (non-COVID-19) Vs Healthy kidney	Total	214
	Up	132
	Down	82
FISH positive Kidney Vs ATI kidney (non-COVID-19)	Total	429
	Up	219
	Down	210
FISH negative Kidney Vs ATI kidney (non-COVID-19)	Total	0
	Up	0
	Down	0
FISH positive Kidney Vs FISH negative Kidney	Total	451
	Up	240
	Down	211

Hantavirus Vs Healthy kidney	Total	935
	Up	594
	Down	341
Hantavirus Vs FISH positive Kidney	Total	1110
	Up	629
	Down	481

Figure S4

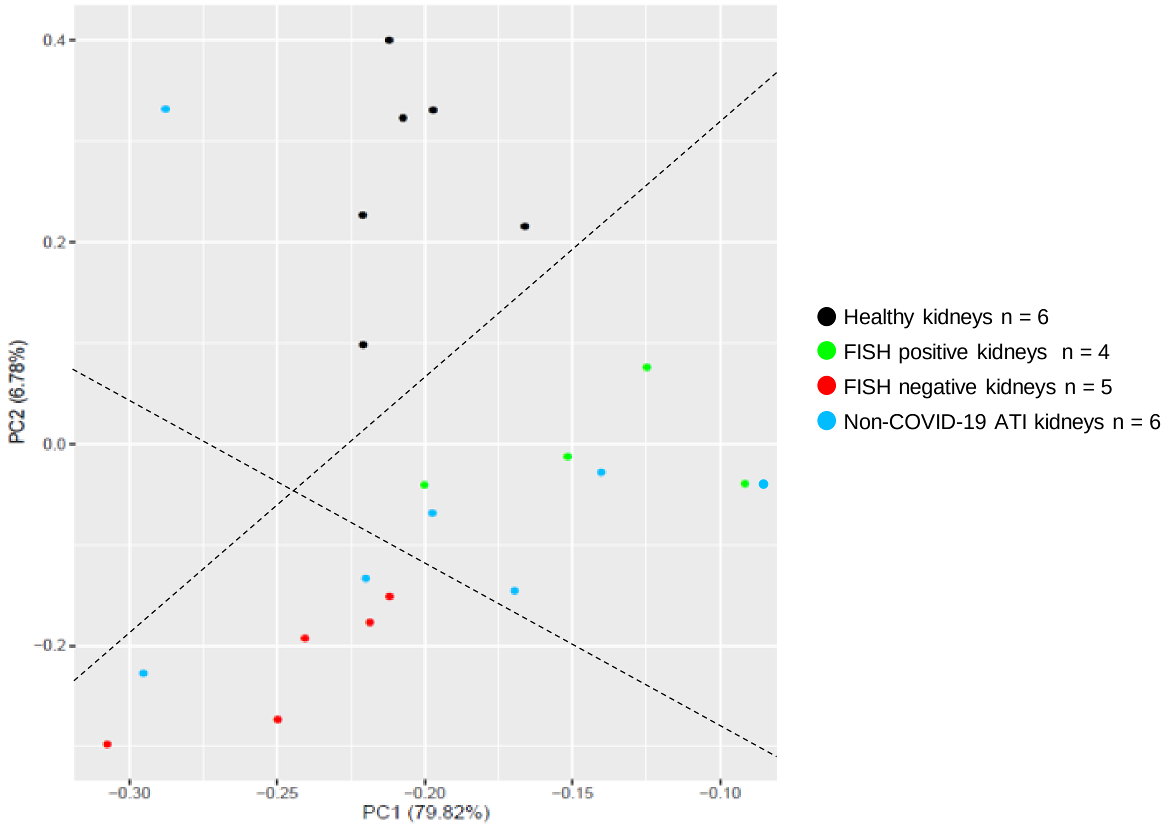

0.4
0.2
0.0
-0.2
PC2 (6.78%)
-0.30
-0.25
-0.20
-0.15
-0.10
PC1 (79.82%)
Healthy kidneys n = 6
FISH positive kidneys n = 4
FISH negative kidneys n = 5
Non-COVID-19 ATI kidneys n = 6

Figure S5

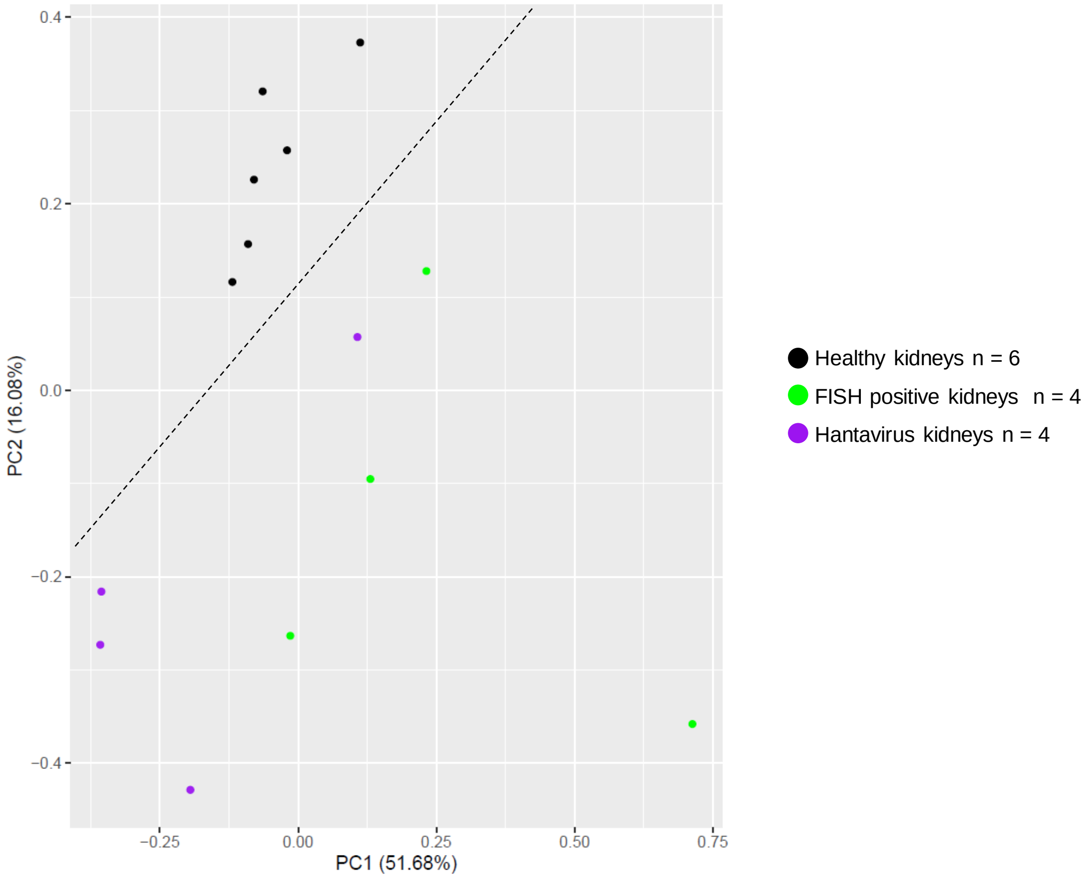
0.4
0.2
0.0
−0.2
−0.4
PC2 (16.08%)
−0.25
0.00
0.25
0.50
0.75
PC1 (51.68%)
Healthy kidneys n = 6
FISH positive kidneys n = 4
Hantavirus kidneys n = 4

Figure S6

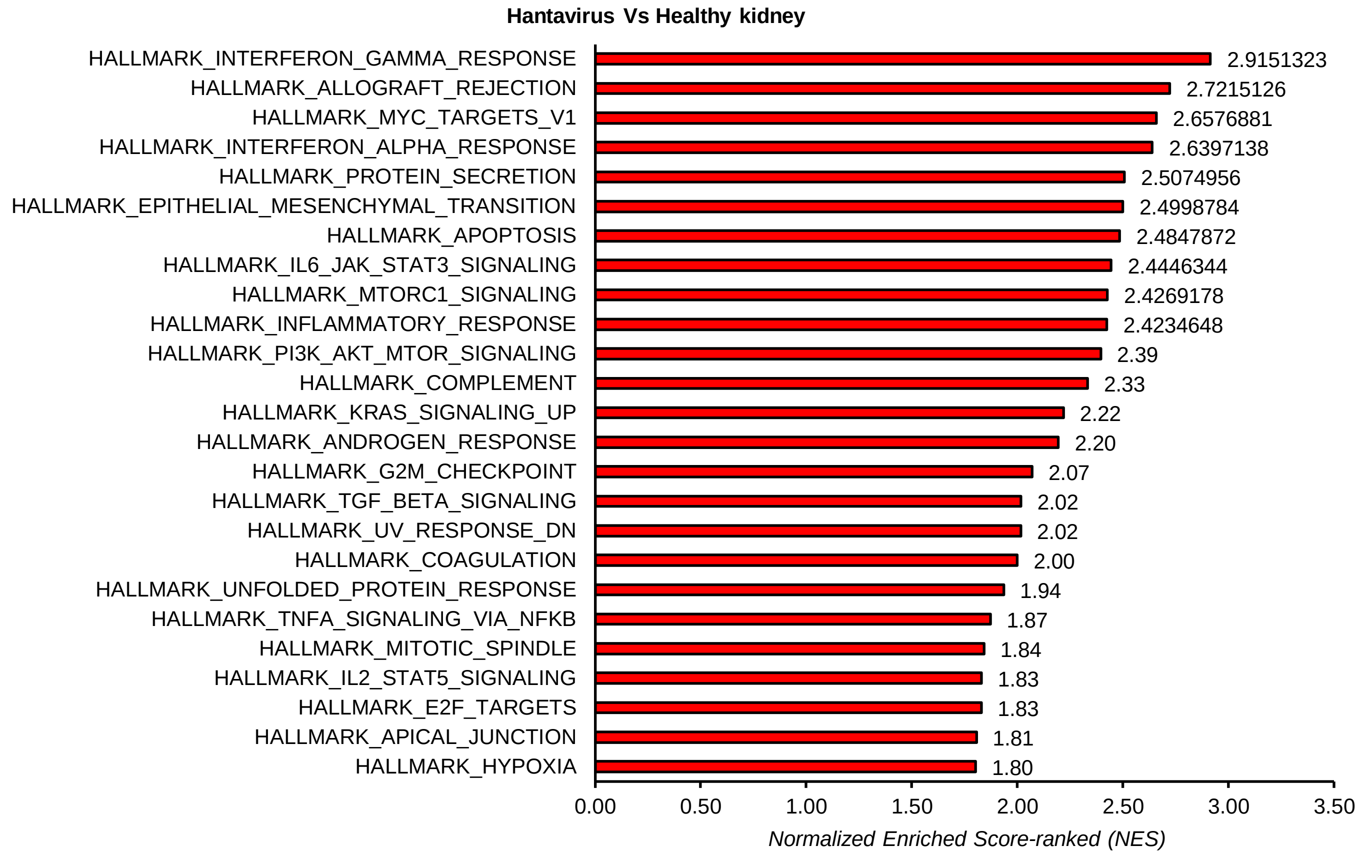

Hantavirus Vs Healthy kidney
HALLMARK_INTERFERON_GAMMA_RESPONSE 2.9151323
HALLMARK_ALLOGRAFT_REJECTION 2.7215126
HALLMARK_MYC_TARGETS_V1 2.6576881
HALLMARK_INTERFERON_ALPHA_RESPONSE 2.6397138
HALLMARK_PROTEIN_SECRETION 2.5074956
HALLMARK_EPITHELIAL_MESENCHYMAL_TRANSITION 2.4998784
HALLMARK_APOPTOSIS 2.4847872
HALLMARK_IL6_JAK_STAT3_SIGNALING 2.4446344
HALLMARK_MTORC1_SIGNALING 2.4269178
HALLMARK_INFLAMMATORY_RESPONSE 2.4234648
HALLMARK_PI3K_AKT_MTOR_SIGNALING 2.39
HALLMARK_COMPLEMENT 2.33
HALLMARK_KRAS_SIGNALING_UP 2.22
HALLMARK_ANDROGEN_RESPONSE 2.20
HALLMARK_G2M_CHECKPOINT 2.07
HALLMARK_TGF_BETA_SIGNALING 2.02
HALLMARK_UV_RESPONSE_DN 2.02
HALLMARK_COAGULATION 2.00
HALLMARK_UNFOLDED_PROTEIN_RESPONSE 1.94
HALLMARK_TNFA_SIGNALING_VIA_NFKB 1.87
HALLMARK_MITOTIC_SPINDLE 1.84
HALLMARK_IL2_STAT5_SIGNALING 1.83
HALLMARK_E2F_TARGETS 1.83
HALLMARK_APICAL_JUNCTION 1.81
HALLMARK_HYPOXIA 1.80
0.00 0.50 1.00 1.50 2.00 2.50 3.00 3.50
Normalized Enriched Score-ranked (NES)

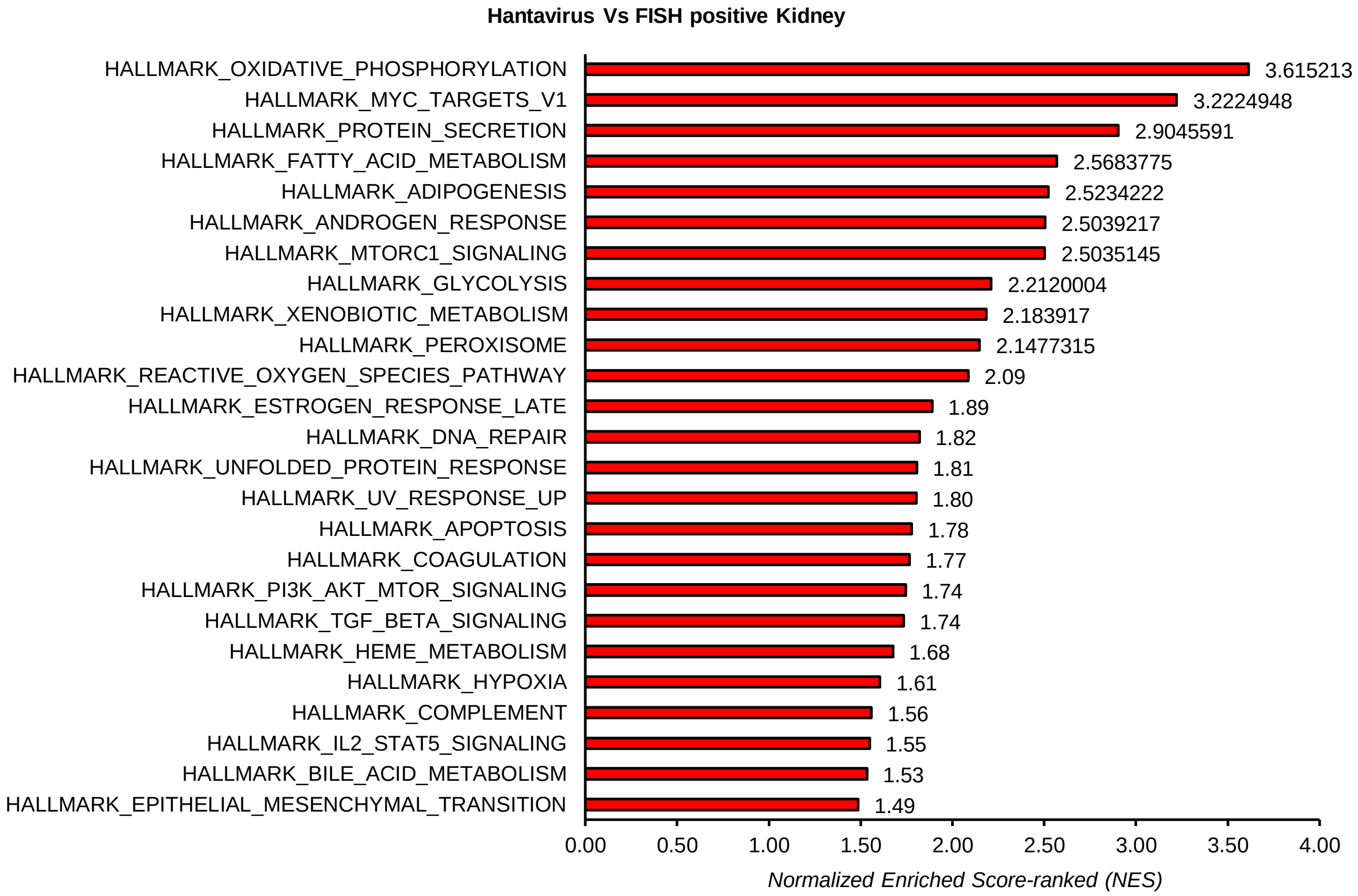

Hantavirus Vs FISH positive Kidney
HALLMARK_OXIDATIVE_PHOSPHORYLATION 3.615213
HALLMARK_MYC_TARGETS_V1 3.2224948
HALLMARK_PROTEIN_SECRETION 2.9045591
HALLMARK_FATTY_ACID_METABOLISM 2.5683775
HALLMARK_ADIPOGENESIS 2.5234222
HALLMARK_ANDROGEN_RESPONSE 2.5039217
HALLMARK_MTORC1_SIGNALING 2.5035145
HALLMARK_GLYCOLYSIS 2.2120004
HALLMARK_XENOBIOTIC_METABOLISM 2.183917
HALLMARK_PEROXISOME 2.1477315
HALLMARK_REACTIVE_OXYGEN_SPECIES_PATHWAY 2.09
HALLMARK_ESTROGEN_RESPONSE_LATE 1.89
HALLMARK_DNA_REPAIR 1.82
HALLMARK_UNFOLDED_PROTEIN_RESPONSE 1.81
HALLMARK_UV_RESPONSE_UP 1.80
HALLMARK_APOPTOSIS 1.78
HALLMARK_COAGULATION 1.77
HALLMARK_PI3K_AKT_MTOR_SIGNALING 1.74
HALLMARK_TGF_BETA_SIGNALING 1.74
HALLMARK_HEME_METABOLISM 1.68
HALLMARK_HYPOXIA 1.61
HALLMARK_COMPLEMENT 1.56
HALLMARK_IL2_STAT5_SIGNALING 1.55
HALLMARK_BILE_ACID_METABOLISM 1.53
HALLMARK_EPITHELIAL_MESENCHYMAL_TRANSITION 1.49
0.00 0.50 1.00 1.50 2.00 2.50 3.00 3.50 4.00
Normalized Enriched Score-ranked (NES)

Figure S7

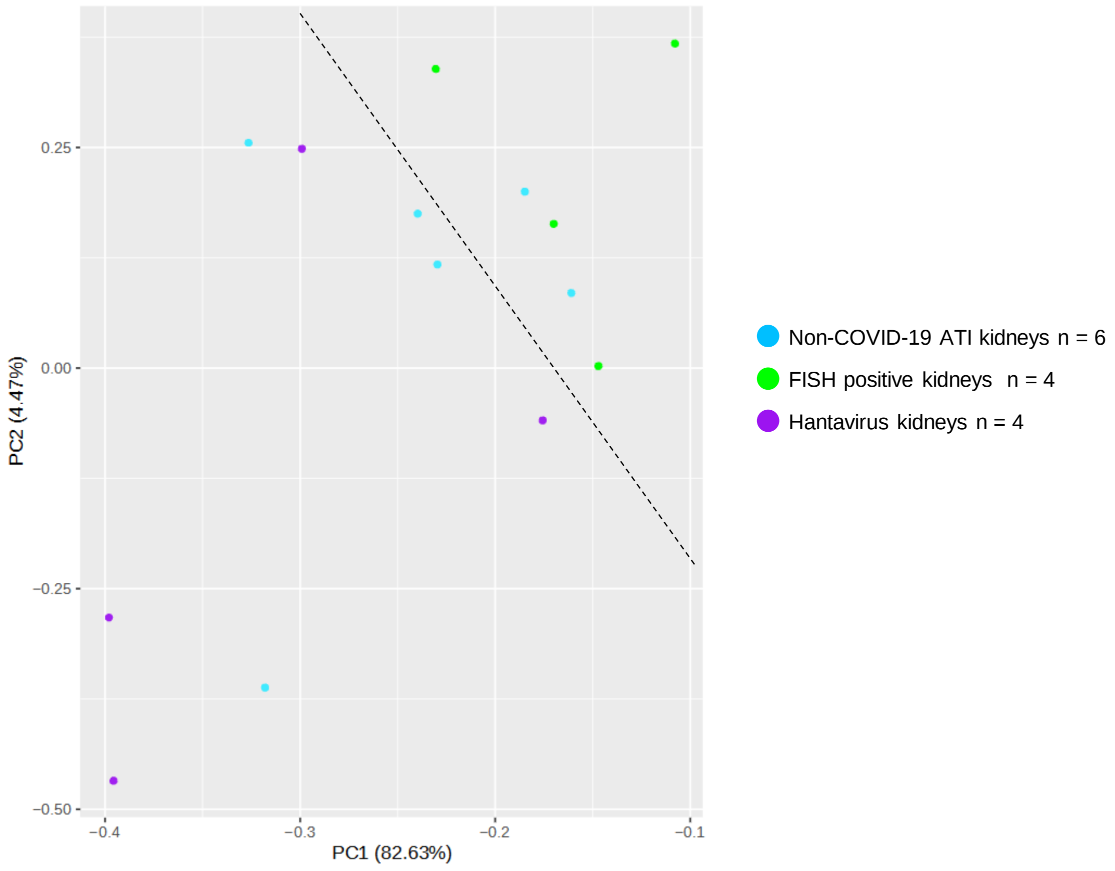
0.25
0.00
−0.25
−0.50
PC2 (4.47%)
−0.4
−0.3
−0.2
−0.1
PC1 (82.63%)
Non-COVID-19 ATI kidneys n = 6
FISH positive kidneys n = 4
Hantavirus kidneys n = 4

Figure S8

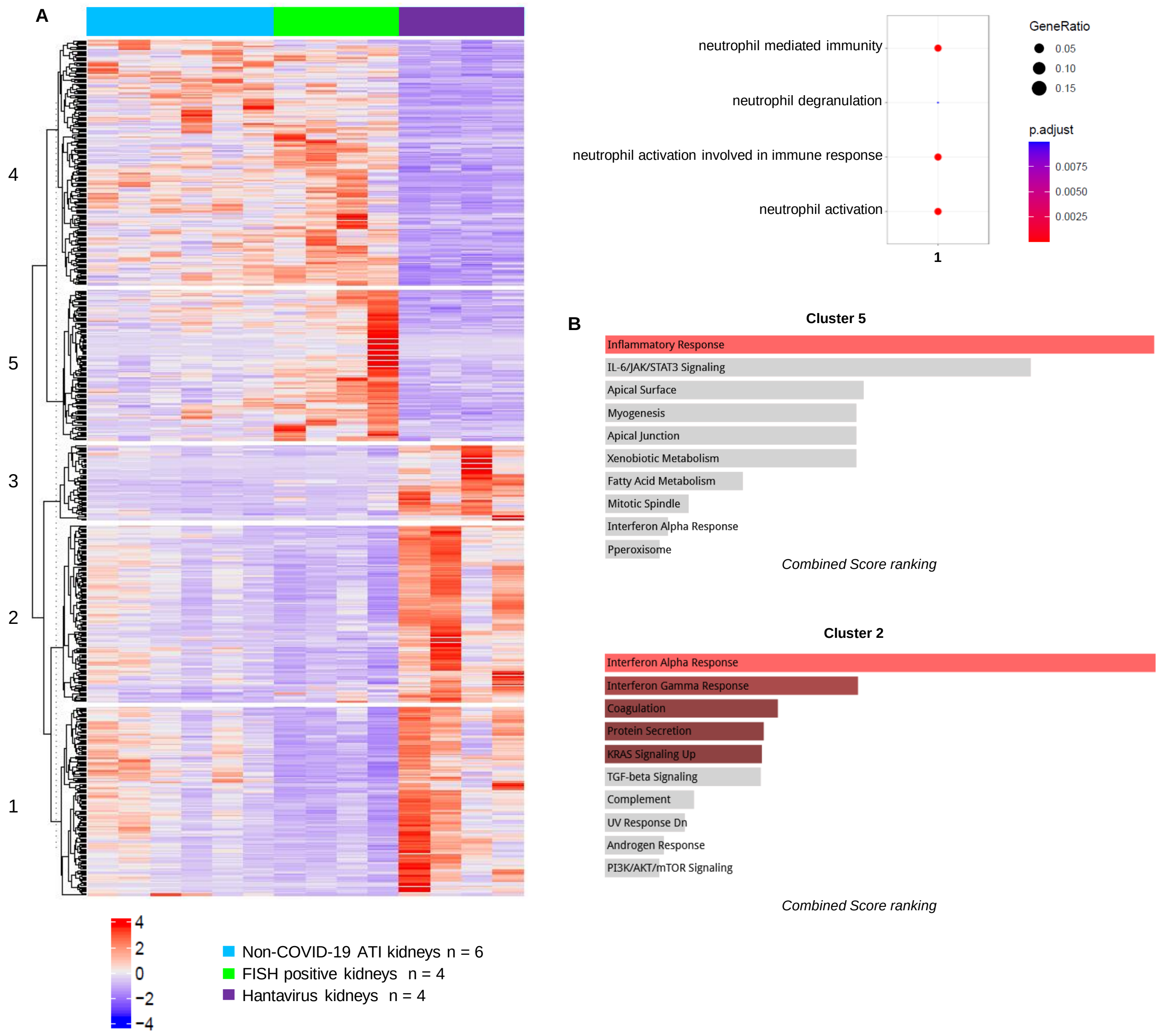
A
4
5
3
2
1
4
2
0
−2
−4
Non-COVID-19 ATI kidneys n = 6
FISH positive kidneys n = 4
Hantavirus kidneys n = 4
neutrophil mediated immunity
neutrophil degranulation
neutrophil activation involved in immune response
neutrophil activation
1
GeneRatio
0.05
0.10
0.15
p.adjust
0.0075
0.0050
0.0025
B
Cluster 5
Inflammatory Response
IL-6/JAK/STAT3 Signaling
Apical Surface
Myogenesis
Apical Junction
Xenobiotic Metabolism
Fatty Acid Metabolism
Mitotic Spindle
Interferon Alpha Response
Pperoxisome
Combined Score ranking
Cluster 2
Interferon Alpha Response
Interferon Gamma Response
Coagulation
Protein Secretion
KRAS Signaling Up
TGF-beta Signaling
Complement
UV Response Dn
Androgen Response
PI3K/AKT/mTOR Signaling
Combined Score ranking

Figure S9
